# Identification of disease-stage therapeutic responses of mesenchymal stromal cells retrieved from murine osteoarthritic joints

**DOI:** 10.3389/fcell.2025.1521437

**Published:** 2025-03-26

**Authors:** Ana Ivanovska, Patrizio Mancuso, Amy Burke, Conor Hennessy, Swarna Raman, Claire Dooley, Steven McLoughlin, Georgina Shaw, Eka Mukeria, Jamie Reilly, Aisling O’Brien, Thomas Ritter, Aideen E. Ryan, Raj Kamath, Marc C. Levesque, Deborah Van Riet, Karen English, Ian Hawthorne, Brian Johnstone, Derek W. Morris, Frank Barry, J. Mary Murphy

**Affiliations:** ^1^ School of Medicine, Regenerative Medicine Institute (REMEDI), University of Galway, Galway, Ireland; ^2^ Centre for Research in Medical Devices (CÚRAM), University of Galway, Galway, Ireland; ^3^ AbbVie Bioresearch Center, Worcester, MA, United States; ^4^ Cellular Immunology Lab, Department of Biology, Kathleen Lonsdale Institute for Human Health Research, Maynooth University, Maynooth, Ireland; ^5^ Department of Orthopaedics and Rehabilitation, Oregon Health & Science University, Portland, OR, United States; ^6^ Discipline of Biochemistry, Center for Neuroimaging, Cognition and Genomics (NICOG), University of Galway, Galway, Ireland

**Keywords:** mesenchymal stromal cells, osteoarthritis, murine model, disease-stage response, chondroprogenitor cells

## Abstract

**Objective:**

Osteoarthritis (OA) is a widespread and debilitating joint disease characterized by synovial inflammation, cartilage degeneration, and chronic joint pain. Mesenchymal stromal cells (MSCs) have shown therapeutic efficacy for many diseases with a strong inflammatory profile, including OA. However, the disease-specific mechanisms of action underpinning the effects of post-local MSC delivery remain unaddressed. In this study, we aimed to characterize the disease-induced profile of MSCs following exposure to the *in vivo* osteoarthritis environment.

**Methods:**

Murine syngeneic GFP + bone marrow-derived MSCs (BM-MSCs) were delivered *via* intra-articular injection in a mouse collagenase-induced osteoarthritis (CIOA) model (n = 8). BM-MSCs were retrieved by cell sorting on days 14 and 56, following whole mouse knee digestions. The retrieved cells were expanded in culture and characterized based on their phenotype, immunomodulatory effects on lymphocytes and macrophages, and transcriptomic profile.

**Results:**

Retrieved BM-MSCs (1.33%) had minimal effects on lymphocyte proliferation but induced macrophage anti-inflammatory activity. Surviving retrieved BM-MSCs activated various pathways, with their secretome impacting immune system regulation and extracellular matrix organization, correlating with the disease stage. Data comparing the transcriptomic profiles of retrieved and *in vitro-*licensed BM-MSCs suggested a chondroprogenitor profile and identified BRINP3 as a novel factor in MSC function for potential OA modulation.

**Conclusion:**

The beneficial effects of BM-MSCs in OA post-local delivery could be attributed to a specific subset of cells able to resist the micro-inflammatory milieu and contribute to cartilage healing and suppression of associated synovial inflammation. Furthermore, data suggest a paradigm of environmentally guided plasticity associated with MSCs upon local delivery in both early and late OA.

## 1 Introduction

Osteoarthritis (OA) is a widespread, debilitating, and chronic joint disease characterized by inflammation and degradation of articular cartilage (AC). It is a major global contributor to disability, becoming more prevalent in aging populations with a severe impact on the quality of life (Das and Farooqi, 2008; Yucesoy et al., 2015). It also represents a significant economic burden in terms of loss of income and healthcare delivery (Xie et al., 2016). Despite the global burden, OA is still an untreatable disease with no pharmacological, biological, or medical intervention that reverses the degradative course. As such, there is a clear need for disease-modifying interventions that can slow progressive joint destruction, reduce pain, restore function, and delay or eliminate the need for joint replacement.

AC injuries are difficult to treat with slow cellular turnover, contributing to limited intrinsic reparative capacity (Sophia Fox et al., 2009). Endogenous mesenchymal stem/stromal cells (MSCs), localizing to synovium, synovial fluid, fat pad, the superficial zone of AC, perichondral groove of Ranvier, and meniscal red zone, can be recruited in response to OA, traumatic injuries, and aging. However, due to age-related and disease-induced changes, the capacity of endogenous MSCs to induce significant disease amelioration is limited (Mantripragada et al., 2019). The ability of MSCs to respond to the injured joint environment *in vivo* by secreting reparative factors was first shown in a caprine model of OA. In this model, cells injected into joints subjected to meniscectomy and anterior cruciate ligament destabilization engrafted onto the synovium and the surface of the regenerated, host cell-derived medial meniscus (Murphy et al., 2003). Although engrafted MSCs survived for at least 6 weeks post intra-articular (IA) injection, they did not differentiate into chondrocytes *in vivo* but significantly impeded the progression of osteoarthritic symptoms. In addition, IA administration of murine MSCs resulted in minimal engraftment in arthritic knee joints in multiple studies (8) with a decreased expression of inflammatory mediators in the synovium described in the collagenase-induced OA model (CIOA) in mice (Ter Huurne et al., 2012; Schelbergen et al., 2014). Associations between apoptosis and MSC-based immunosuppressive effects were first shown in a graft-versus-host disease model, where intravenously introduced MSCs underwent apoptosis; apoptotic cells were phagocytosed by macrophages with induced release of indoleamine-pyrrole 2,3-dioxygenase (IDO), promoting the resolution of inflammation (Galleu et al., 2017).

We hypothesized that MSC-induced OA modulation following local delivery is initially associated with apoptosis-mediated immune reprogramming through phagocytosis by synovial lining macrophages, and surviving MSCs were licensed through exposure to the inflamed OA joint environment, enabling them to respond by molecular reprogramming specific to the “injured” environment. To investigate the fate and molecular mechanisms underpinning MSC therapeutic effects in OA, syngeneic eGFP BM-MSCs were retrieved from whole joint digests using fluorescence-activated cell sorting (FACS) after local IA delivery in a murine OA model induced by collagenase type VII. Molecular and phenotypic characterization of the surviving implanted cells was performed to identify mechanisms underpinning 1) the MSC therapeutic response after joint injury and 2) the molecular response to the established OA environment.

## 2 Materials and methods

### 2.1 Isolation of murine BM-MSCs

Wild-type (wt) BM-MSCs were isolated from C57BL/6 mice (Charles River Laboratories, Margate, Kent, United Kingdom). GFP + BM-MSCs were isolated from C57BL/6-Tg (UBC-GFP) 30Scha/J mice (Jackson Laboratory, Bar Harbor, ME, United States) (Da Silva Meirelles and Nardi, 2003). All animals (male, 8–12 weeks) were maintained on a 12-h light/dark cycle with *ad libitum* access to standard laboratory chow and water. All procedures were approved by the Animal Care and Research Ethics Committee (ACREC) of the National University of Ireland, Galway, and conducted under licenses issued by the Health Products Regulatory Authority (HPRA), Dublin, Ireland.

Following euthanasia, femurs and tibias were dissected, and the flushed BM was filtered using a 40-μm cell strainer and centrifuged at 400x g for 10 min. The obtained pellet was stained with trypan blue (Sigma-Aldrich), and the cells were cultured at a density of 2 × 10^6^/cm^2^ in a humidified 5% CO_2_ incubator at 37°C for 3–5 days. The medium was changed to remove non-adherent cells, and BM-MSCs were fed every 3–4 days until they reached ∼80% confluence. At this point, they were detached using trypsin/EDTA (ethylenediaminetetraacetic acid) (Gibco, Thermo Fisher Scientific) and re-plated at a density of 5 × 10^3^/cm^2^.

### 2.2 Intra-articular injection

Experimental CIOA was initiated bilaterally in the knee joints of C57BL/6 mice by IA injection of 1 unit of purified bacterial type VII collagenase (Sigma-Aldrich) in 7 μL of physiological saline. The control group (sham) received saline only, with n = 6 mice per each group. IA injections were performed under isoflurane-induced anesthesia using a 10-μL syringe (Hamilton Company, Nevada, United States) with a 27G needle inserted laterally to prevent patellar ligament damage. Two collagenase treatments were carried out on days 0 and 2. On day 7, the mice received an IA injection of 2 × 10^5^ GFP + BM-MSCs suspended in 7 µL of α-MEM (Gibco, Thermo Fisher Scientific). All animals were included in the analysis.

### 2.3 Digestion of whole mouse knees

C57BL/6 mice were euthanized 12 h and 72 h post-BM-MSC delivery by CO_2_ overdose, followed by cervical dislocation. Whole limbs were removed and cleaned of muscle tissue while preserving the synovium. The AC was exposed by the dissection of the synovial membrane along with the frontal cruciate ligament. The exposed tissue was incubated with 100 µL of 1 mg/mL collagenase type I (*Clostridium histolyticum*, Sigma-Aldrich C0130). After 3 h, the released cell suspension was collected, and fresh collagenase was added for an additional 3 h. Knee digests from the same experimental groups were pooled.

### 2.4 Cell sorting

For FACS-coupled cell sorting of the retrieved BM-MSCs, the cell suspensions were washed in FACS buffer (PBS, 1% FBS, 2 mM EDTA, and 25 mM HEPES) and sorted using FACS Aria (BD Biosciences). Controls for establishing gating included a non-injected knee digest and a knee digest spiked with 1 × 10^5^ GFP-MSCs. DRAQ7 viability dye was used to exclude dead cells. Receiving tubes were pre-coated with FBS and filled with 1 mL of the MSC culture medium. Retrieved BM-MSCs were cultured in this medium, fed every 3–4 days, and passaged at approximately 80% confluence until passage 2. Cell-conditioned medium (CM) was collected at after 48 h of passage 2 culture, and cell debris was removed by centrifugation.

### 2.5 Immunophenotyping of naïve and retrieved BM-MSCs

For immunophenotyping, cells were stained using antibodies (Supplementary Table S1) targeting CD105, CD106, CD146, CD44, and Ly-6A/E (Sca-1) (BioLegend); CD140b and CD29 (eBioscience); and CD90.2, CD11b, and CD45 (BD Pharmingen) in FACS buffer (PBS with 2% FBS and 0.05% sodium azide (NaN3) at 4°C in the dark for 30 min. Isotype controls were used to differentiate non-specific background signals. All samples were analyzed on a FACS Canto A (BD Biosciences) using FACS Diva software (BD Biosciences).

### 2.6 ELISA assays for cytokine quantification

Next, we investigated the immunomodulatory ability of retrieved cells from SHAM and CIOA joints (Section 1.2 of Supplementary Material and Methods). Commercially available ELISA kits were used to determine the concentration of soluble cytokines in MSC and BMDM culture media, as per the manufacturer’s instructions. For IL-10, IL-12 p70, and TNF-α, DuoSet ELISAs (R&D systems DY417, DY419, and DY410) were used with minor modifications to the manufacturer’s protocol. These cytokines have been previously identified in the osteoarthritic synovial fluid of human patients with knee OA (Sohn et al., 2012) and validated in a preliminary study aimed at identifying the *in vivo* inflammatory profile of the CIOA model (Section 1.3 of Supplementary Material and Methods; Supplementary Table S5). In brief, 96-well flat-bottom plates were coated with rat anti-mouse IL-10/IL-12 p70/TNF-α capture antibodies, diluted in PBS, and incubated overnight at RT. Following sample incubation, plates were washed, and biotinylated goat anti-mouse IL-10/IL-12 p70/TNF-α detection antibodies diluted in reagent diluent were added. After incubation, plates were washed, and streptavidin-horseradish peroxidase (1:200 dilution) was added. Plates were incubated and washed, and the TMB/E substrate solution (Merck Millipore) was added, followed by a stop solution (2N H2SO4). Optical density was determined using a Wallac 1420 Victor 3 plate reader set to 540/450 nm. For PGE2 quantification, the Parameter Assay Kit (R&D systems KGE004) was used.

### 2.7 Nitric oxide assay

The amount of nitric oxide (NO) present in cell culture supernatants was measured using Griess assays. In brief, Griess reagent, consisting of H_2_O, 1% sulphanilamde, 0.1% naphthylethylene diamine, and 5% phosphoric acid, was added in a 1:1 ratio to samples or standards (sodium nitrite, NaNO_2_, 1,000 to 15.625 μM in culture medium), and incubated for 10 min at RT in the dark. Optical density was determined using a Wallac 1420 Victor 3 plate reader set to 540 nm.

### 2.8 Retrieval and molecular analysis of BM-MSCs

GFP + BM-MSCs from sham or CIOA knee joints were sorted directly into ice-cold PBS, snap-frozen, and stored at −80°C. Control, non-injected GFP-MSCs (5 × 10^3^) treated with 50 ng/mL of IL6 or a combination of 50 ng/mL IL6, IFN-γ, and monocyte chemoattractant protein-1 (MCP-1) (triple licensing cocktail) were also included for analysis. RNA was isolated from all samples using the RNeasy^®^ Micro RNA Isolation Kit (QIAGEN), following the manufacturer’s instructions and making the following adjustments due to the low cell numbers available. RNA was quantified using the RNA 6000 Pico Kit and analyzed using the Bioanalyzer 2100 (both from Agilent Technologies). First-strand cDNA synthesis was performed using the SMART-Seq^®^ v4 Ultra^®^ Low Input RNA Kit (TaKaRa Bio USA, INC.), and cDNA quality was determined using bioanalyzer high-sensitivity DNA chips. Following quality control using the bioanalyzer, tagmentation was performed using the Nextera XT DNA Sample Preparation Kit and Nextera XT DNA Index Kit (both from Illumina). Once tagged, samples were normalized and pooled. Sample quality control was performed prior to sequencing at the Genomics Core Technology Unit (Queen’s University Belfast, Northern Ireland), and FASTQ files were generated for further bioinformatics analysis (Section 1.5 of Supplementary Materials and Methods).

### 2.9 Immunofluorescence staining validation

Murine joints were dissected, fixed in 10% formalin for 1 h, and decalcified in 10% EDTA in PBS for 2 weeks at 4°C. E14–E15 embryonic mouse limb sections (Murphy et al., 1999) and chondrogenic pellets from human BM-MSCs and articular cartilage progenitor cells (ACPs) (Anderson et al., 2018) were analyzed. For the latter, 1 × 10^5^ cells/pellet were processed for chondrogenic differentiation induced by 10 ng/mL TGF-β3 (Peprotech) and 100 ng/mL BMP-2 (Peprotech) at 2% O_2_ and 5% CO_2_ for 28 days. The pellets and decalcified mouse joints were washed, fixed in 10% formalin, dehydrated (Leica ASP300 S), paraffin embedded, sectioned, and mounted for IF staining. Fixed or fixed and decalcified sections were heated at 60°C for 1 h and rehydrated in graded ethanol (100%–75%) before antigen retrieval using 1 mg/mL pronase in PBS for 5–10 min and blocked with 10% goat serum for 1 h at room temperature. The primary antibodies anti-FAM5C (ab254837, Abcam), anti-lubricin/MSF (ab254937), and anti-Collagen II (ab185430) were applied overnight (1:100–1:200) at 4°C, and the signal was visualized post 1 h incubation with the corresponding secondary fluorochrome-conjugated antibodies (Anti-rabbit IgG (H + L), F (ab’) 2 Fragment–Alexa Fluor 488, Alexa Fluor 555; and Anti-mouse IgG (H + L), F (ab’) 2 Fragment–Alexa Fluor 555, Cell Signaling Technology) at room temperature with DAPI-mounting medium used for nuclei staining. BRINP3 antibody specificity was validated in human teratomas (Supplementary Figure S11) and rabbit isotype control IgG (ab37415, Abcam) was used in tested samples to confirm BRINP3 signal specificity. Confocal microscopy images were taken using the Olympus Fluoview FV3000 Microscope (FV31S-SW software).

### 2.10 Statistical analyses

Statistical analysis was performed using GraphPad Prism version 5. Significance was assessed using one-way or two-way ANOVA, followed by a Tukey test. Error bars represent the mean ± standard error of the mean (SEM) of biological replicates, and p-values ≤0.05 were considered statistically significant. Statistical significance was assessed. When the sample number was n ≤ 3, data were considered biological replicates (different donor), and where biological replicates were not available (n = 1), the mean ± SEM of technical triplicates is shown without statistical analysis.

## 3 Results

### 3.1 Characterization of retrieved vs. naïve BM-MSCs

QDot-labeled syngeneic MSCs (2 × 10^5^) were delivered to CIOA mouse joints by IA injection to quantify cell survival using Cryoviz imaging (Supplementary Figure S1). A single cluster of cells was detected in the joint 1 h post-delivery, with 1.4% of injected cells surviving at 72 h, indicating rapid clearance of the majority of delivered cells. Similarly, retrieval optimization experiments performed in healthy mice (n = 4; 8 knee joints) with 2 × 10^5^ GFP + BM-MSCs delivered *via* bilateral IA injection recovered only 1.627% of the injected cells (Supplementary Figure S2). When these transplanted, retrieved, and sorted GFP + BM-MSCs were cultured, they proliferated with growth kinetics and morphology similar to the control (untransplanted) population. Retrieved cells showed increased clonogenicity as measured by CFU-F activity and demonstrated greater osteogenic and chondrogenic propensity compared to control cells (Supplementary Figure S3). Data indicated the retrieval of a small population of engrafted cells that retained overt characteristics of BM-MSCs.

**FIGURE 1:**
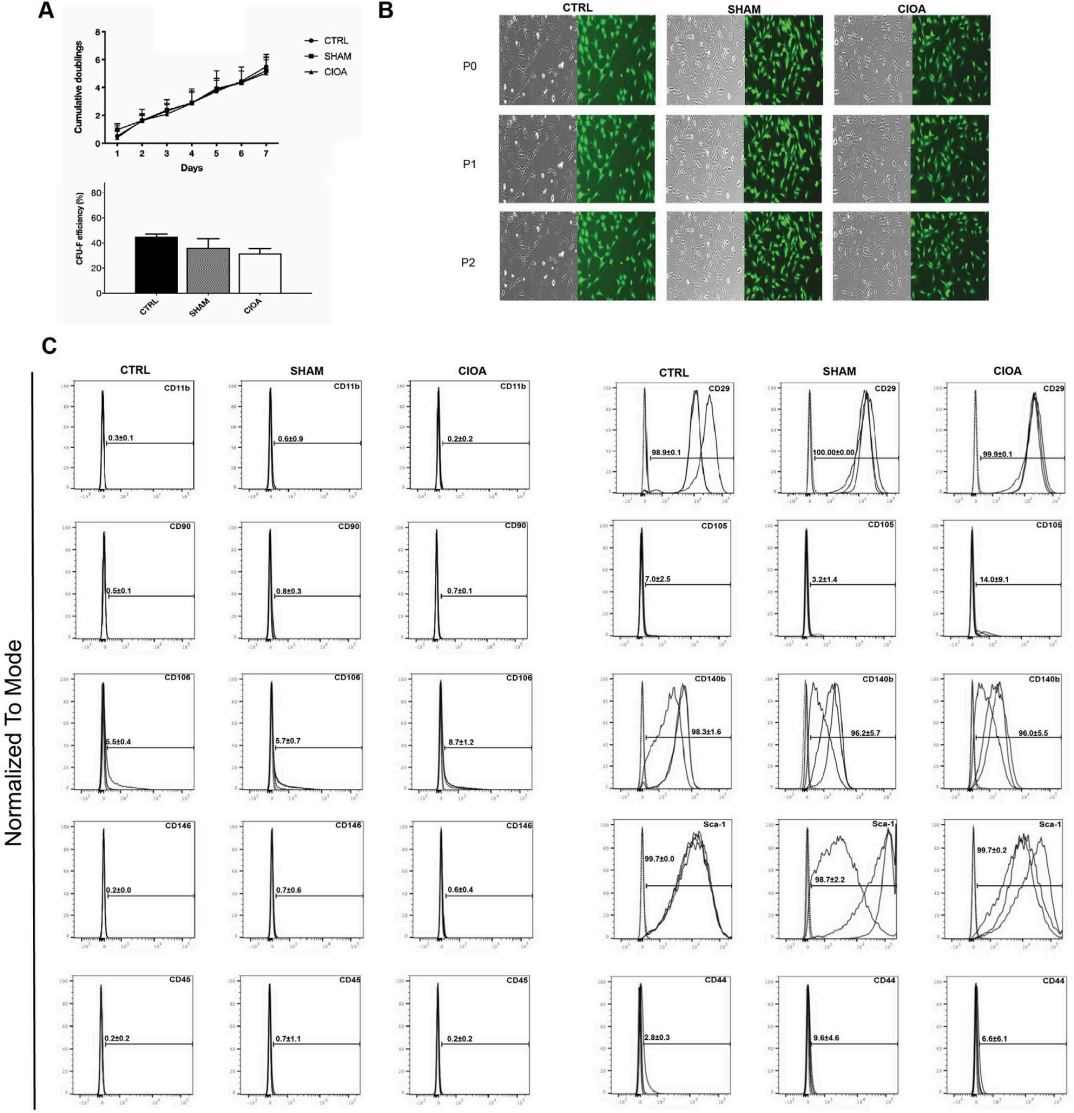
Characterization of BM-MSCs retrieved from the CIOA joint. **(A)** Proliferation of retrieved BM-MSCs and CFU-F efficiency at passage 2 post-retrieval; mean + SEM (n = 3). **(B)** Representative images of sorted cells; bright field (left) and FITC (right); scale bar, 100 µm. **(C)**: Surface marker expression of retrieved MSCs.

Experiments (n = 3) involving the delivery and retrieval of GFP-MSCs in CIOA joints were then assessed. In brief, 2 × 10^5^ BM-MSCs were delivered bilaterally via IA injection to knees previously treated with collagenase Type VII to induce CIOA or saline as a control (sham) (n = 6; 12 knee joints). Three days post-administration, whole joints from both groups were exposed and digested to generate single-cell suspensions. GFP^+^ cells were separated from the suspension by fluorescent cell sorting. Yields varied between 0.01% and 0.332% of 2 × 10^5^ cells injected (Supplementary Table S2). The mean quantitative recovery of cells from OA joints (0.134% of injected cells) was consistently higher than that from sham joints (0.02% of injected cells), suggesting greater persistence of engrafted cells in the OA environment. Retrieved GFP + BM-MSCs formed characteristic colonies in cultures with comparable morphology and proliferation rates to control non-injected MSCs. Control GFP + BM-MSCs (CTRL) and retrieved sham- and CIOA-injected cells were negative for CD45, CD31, and CD11b, with Sca-1, CD29, and CD140b highly expressed. CD105 and CD90 were minimally detected in both cell populations (Figure 1).

### 3.2 Retrieved MSCs exhibit an immunomodulatory effect on T cells and bone marrow-derived macrophages

**FIGURE 2:**
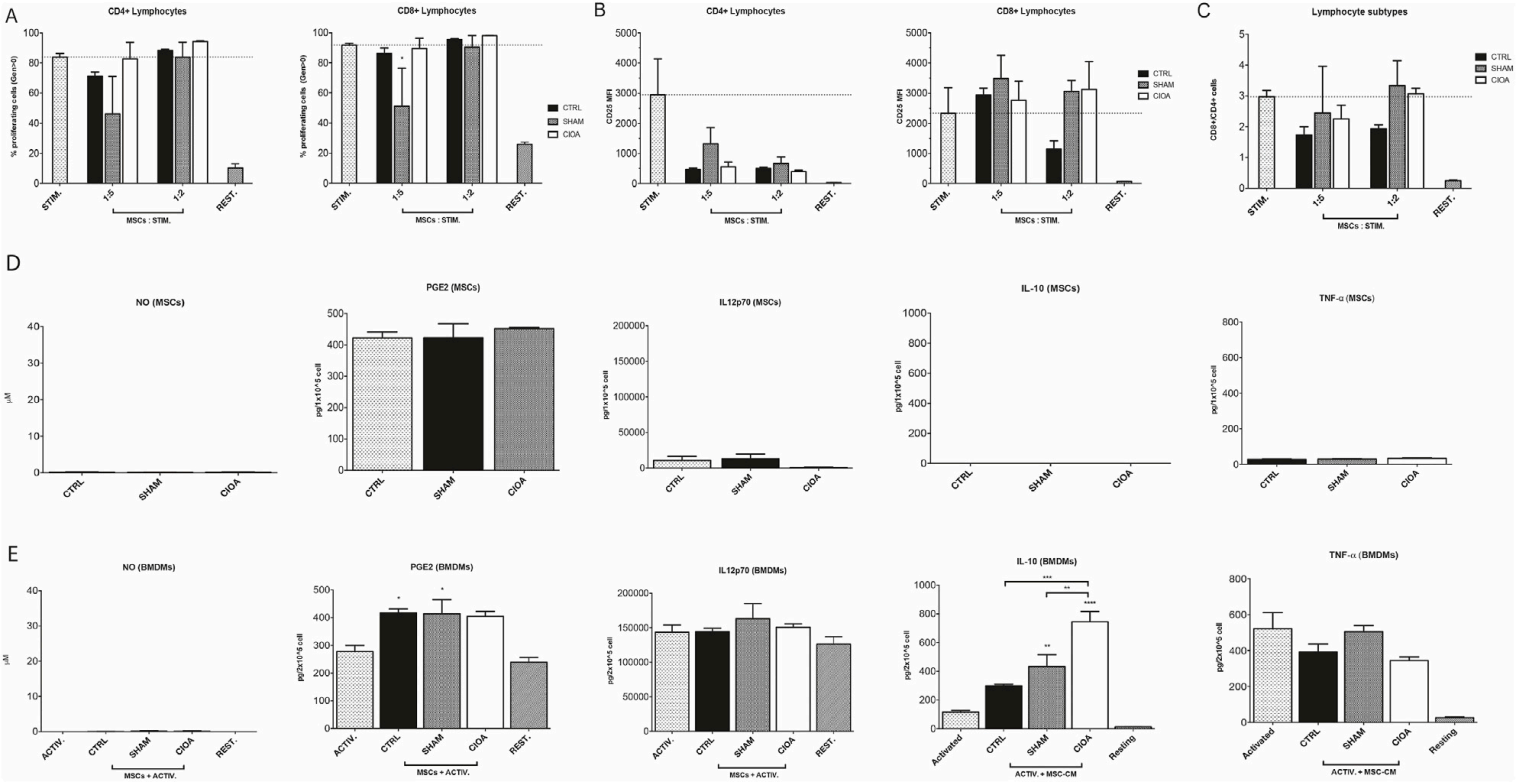
Immunomodulatory potential of retrieved BM-MSCs. **(A)** Proliferation of CD4^+^ and CD8^+^ T cells after the third generation, quantified by CTV staining. **(B)** Expression of CD25 by CD4^+^ and CD8^+^ T cells. **(C)** CD4+/CD8+ T cell relative quantity. Secretion of NO, PGE2, IL12p70, IL-10, and TNF-alpha from **(D)** naïve (CTRL) or retrieved BM-MSCs from the sham and CIOA experimental groups and from **(E)** BMDMs in activated or resting state and/or treated with conditioned media from control, sham, and CIOA BM-MSCs (data were shown as the mean ± SEM (n = 3). *P ≤ 0.05; **P ≤ 0.01; ***P ≤ 0.001; ****P ≤ 0.0001. Statistical significance compared to activated macrophages (ACTIV) as positive control, unless indicated by brackets.

The assessment of immunomodulation by non-injected (Ctrl), sham, and CIOA-retrieved BM-MSCs revealed that MSC populations were unable to inhibit T-cell proliferation in co-culture experiments, except for the inhibition of CD8-positive cells by sham-retrieved BM-MSCs (Figure 2).

Bone marrow derived macrophages (BMDMs) were cultured with CM from control, sham, and CIOA-retrieved BM-MSCs. BMDMs released anti-inflammatory IL-10 in response to LPS-mediated activation; while naive MSC-CM induced low IL-10 levels, CM from SHAM- and CIOA-challenged MSC upregulated IL-10 compared to both macrophages alone and the CTRL-MSC-treated sample. Macrophages exposed to the CIOA environment produced IL10 at levels significantly higher than sham and control BM-MSCs. Immunosuppressive effects of BM-MSCs were supported by decreased, although not significant, TNF-α levels. PGE2 was detected at similar levels in all BM-MSC supernatants, whereas activated and resting BMDMs released PGE2 at comparable levels. Low levels of IL-12 p70 were produced by MSCs, while BMDMs showed high secretion in all groups (Figure 2).

### 3.3 Establishment of the molecular therapeutic phenotype of MSCs in osteoarthritis

Optimal times for cell retrieval to define molecular changes were selected by defining the inflammatory environment in mouse joints and local and systemic macrophage profiles (Supplementary Figure S4). A mouse cytokine panel was designed based on the expression of inflammatory cytokines in the human OA synovial fluid (Sohn et al., 2012) and joint cytokine levels in CIOA, determined 7 to 56 after the first injection of collagenase type VII or saline. Relative levels of selected factors in CIOA versus sham joints demonstrated a pattern of expression where MCP-1, IFNγ, IL-6, RANTES (CCL5 (Chemokine (C-C motif) ligand 5), IL-13, and TNFα showed peak of expression (Supplementary Figure S5). Days 14 and 56 were chosen as selected time points for the injection of MSCs (Ferrao Blanco et al., 2022) for retrieval 12 h and 3 days post-injection to generate the MSC therapeutic signature in OA. For this purpose, additional retrieval experiments were conducted. Supplementary Figure S6, summarizing the numbers of retrieved MSC populations from n = 8 mice (16 knee joints)/per group and timepoint, indicates that significantly more MSCs survived and engrafted in CIOA compared to sham-injected joints, with increased fold recoveries ranging from 1.9 (56 days CIOA, 3-day) to 4.4 fold (14 days CIOA, 0.5-day).

**FIGURE 3:**
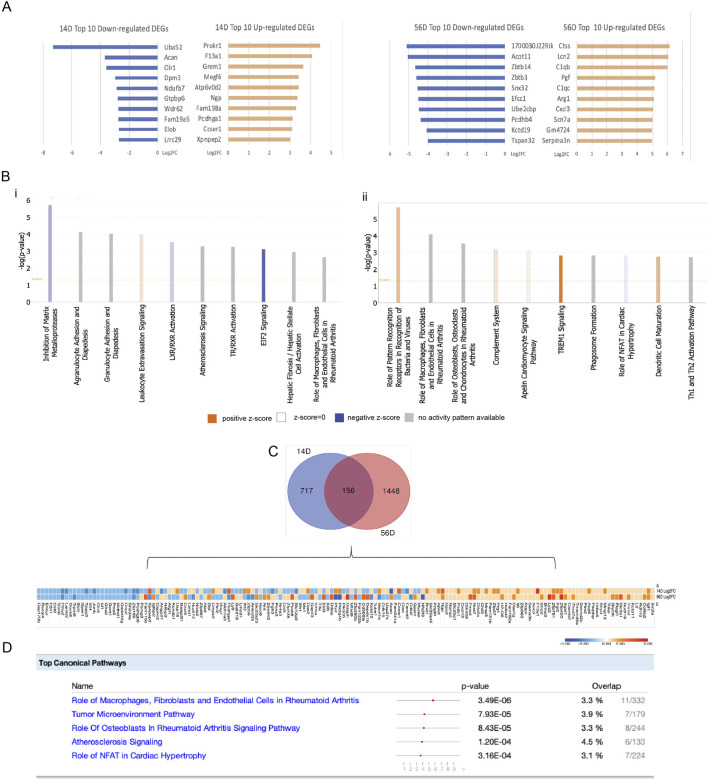
Identification and bioinformatics analysis of DEGs in day 14- and day 56-retrieved cells**. (A)** Top 10 upregulated and downregulated DEGs in 14D and 56D, expressed as log_2_fold change. **(B)** Top 10 canonical pathways identified with the ingenuity pathway analysis on 14D and 56D. **(C)** Overlap of DEGs between 14D and 56D with a representative heatmap of similarly and differently expressed genes. **(D)** Canonical pathways associated with the common DEGs between 14D- and 56D-retrieved BM-MSCs, ranked based on their p-value. For each pathway, the gene overlap compared to the IPA database is shown as numerical value and percentage.

An initial heat map assessment of the RNAseq analysis indicated the extensive gene upregulation and activation of multiple pathways at the 12-h retrieval time point for both early and late CIOA (days 14 and 56, respectively) (Supplementary Figure S7). The 3-day retrieval time point was, therefore, used to identify therapeutic mechanisms. On days 14 and 56, respectively, 878 and 1,606 differentially expressed genes (DEGs) were identified between BM-MSCs retrieved from CIOA and SHAM knee joints. The top 100 upregulated and downregulated genes are listed in Supplementary Tables S6–S9. Figure 3A shows the 10 most significantly upregulated and downregulated genes in each group. Upregulated genes in early OA retrieved cells were associated with promoting cell survival and proliferation, repression of apoptosis through Akt signaling (*PROKR1*), TGF-beta/SMAD activation (*MEGF6*), and autophagy (ATP6V0D2), indicating an association between autophagy and cell survival.

BM-MSCs retrieved at the later OA stage play an immunomodulatory role, interacting with local immune cells through the complement cascade (*C1QB* and *C1QC*), secretion of cytokines and adipokines (*LCN2* and *CXCL3*), growth factors (*PGF*), and cells expressing M2 macrophage markers (*ARG1*).

All DEGs were analyzed to generate canonical pathways associated with early and late OA retrieved cells using ingenuity pathway analysis (IPA) (Figure 3B). In 14D retrieved cells, “leukocyte extravasation signaling” was associated with the highest activation, while “inhibition of matrix metalloproteinases” and “EIF2 signaling” had the lowest activation z-scores. The highest activated pathways in 56D retrieved cells were “TREM1 signaling,” “dendritic cell maturation,” “role of pattern recognition receptors in recognition of bacteria and viruses,” “complement system,” and “apelin cardiomyocite signaling pathway,”, while “role of NFAT in cardiac hypertrophy” had a negative activation z-score (Figure 3B). The cellular molecular fingerprint predicted upstream regulators at both time points. For early OA-retrieved MSCs, IL-1 beta, IFN-gamma, NF-kB, and HIF-1 alpha were the most activated upstream regulators, with TCL1A, STAT3, IL-1 beta, OSM, and CCR3 associated with 56D cells (Supplementary Table S10).

An analysis of DEGs identified in early and late OA-retrieved MSCs identified 156 common elements (Figure 3C), and for these, the top five canonical pathways were identified in IPA (Figure 3D). “Role ofmacrophages, fibroblasts, and endothelial cells in rheumatoid arthritis” showed the highest *p*-value and gene overlap score, indicating a potential immunomodulatory role in cells retrieved from both time points.

All differentially expressed genes were further analyzed for Gene Ontology (GO) enrichment annotations for biological processes, molecular functions, and cellular components. Supplementary Table S11 lists the top five most enriched terms for each ontology in both datasets.

In 14D-retrieved BM-MSCs, the five most enriched GO biological processes were associated with ECM metabolism, regulation of cell migration, and establishment of apical–basal polarity. These terms suggest that MSCs are directly involved in the maintenance of ECM structural integrity, with increased migratory abilities toward injury sites. In 56D-retrieved BM-MSCs, the five most enriched GO biological processes were related to immune processes involving leukocyte aggregation, macrophage differentiation, and T-helper cell differentiation, as well as transport of amino acids. These may suggest that MSCs injected and retrieved in the later CIOA stage exhibit strong immunomodulatory activities through a synergy of mechanisms involved in CD4-positive T-cell and macrophage differentiation. This finding was also supported by the expression of chemokine receptors, promoting interactions with chemokines secreted by the microenvironment. A possible mechanism to counterbalance autophagy-mediated deprivation of essential molecules might be mediated by the upregulation of transport complexes and the enrichment of extracellular amino acid uptake (Zhang et al., 2018).

### 3.4 Disease-related differences in the secretome profile of early and late OA-retrieved MSCs

**FIGURE 4:**
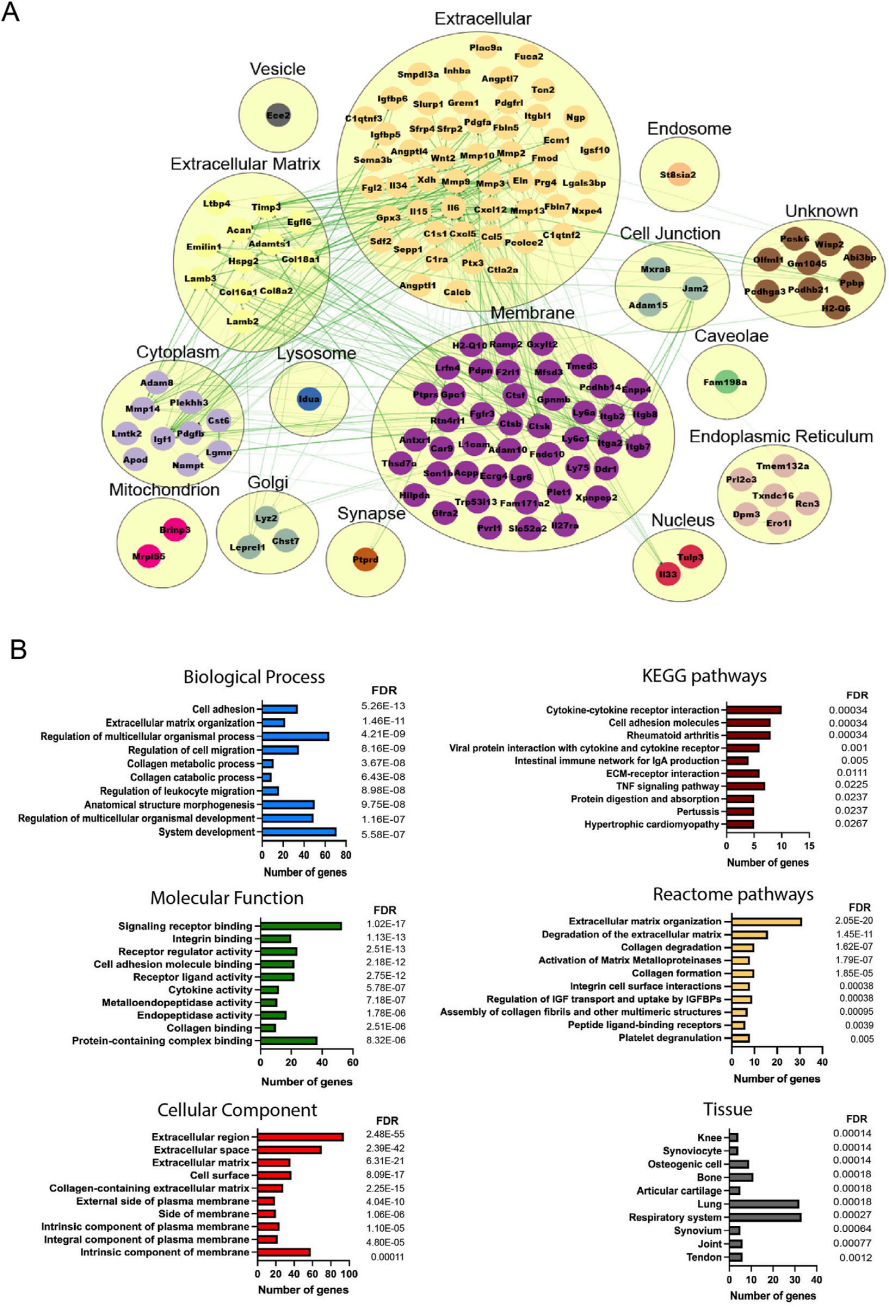
Analysis of the predicted secretome in 14D-retrieved cells**. (A)** Protein–protein interaction (PPI) network visualizing clustering results, where each cluster represents a cellular compartment. Unknown clusters contain elements not mapped to any specific cell location. Green lines represent STRING interaction lines (Zeng et al., 2022). **(B)** Representative enrichment results, including Gene Ontology elements, pathways, and tissues corresponding to the PPI network. Terms are ranked based on the FDR value, and for each term, the number of corresponding genes is represented.

**FIGURE 5:**
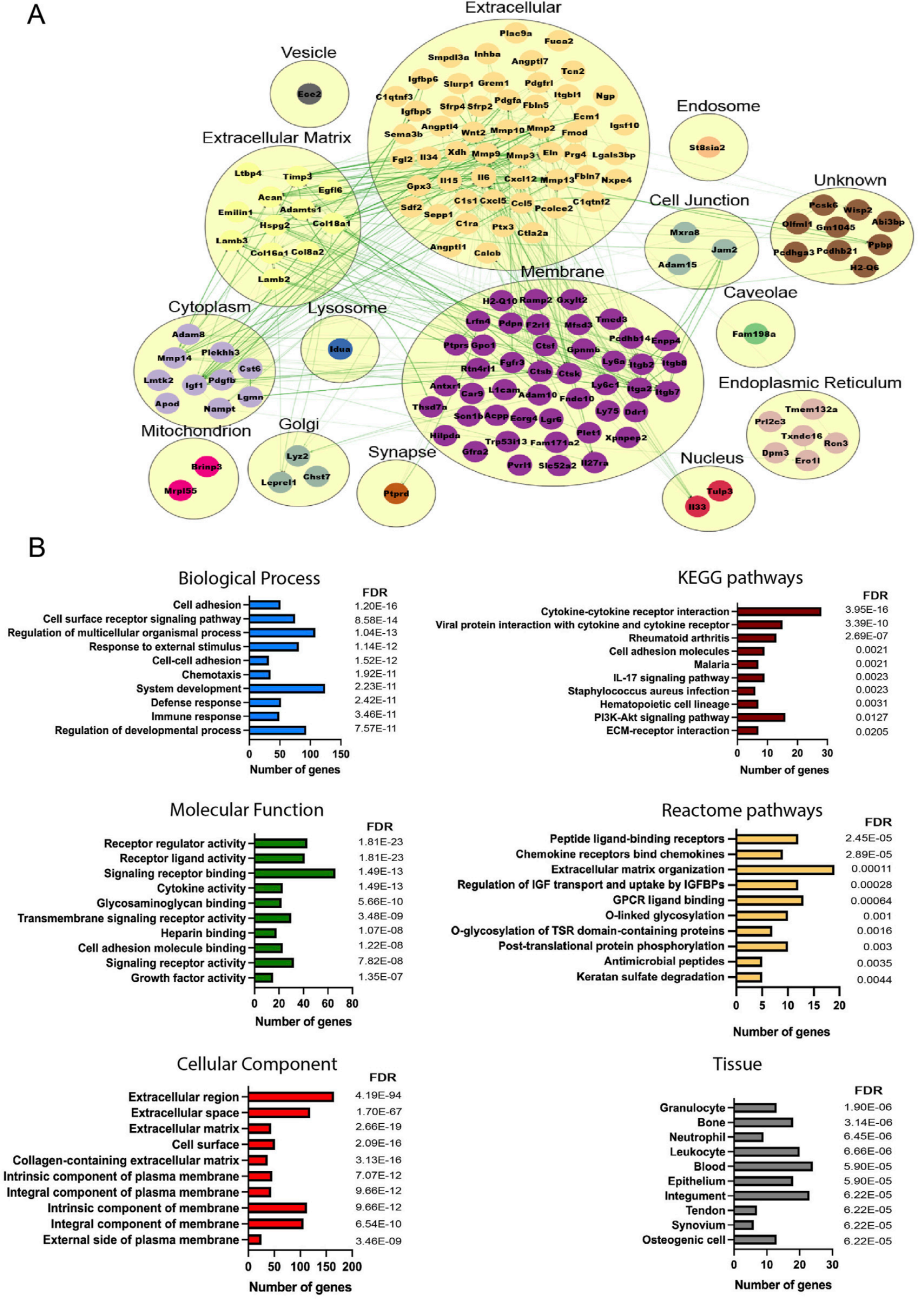
Analysis of the predicted secretome in 56D-retrieved cells**. (A)** PPI network visualizing clustering results, where each cluster represents a cellular compartment. Unknown clusters contain elements not mapped to any specific cell location. Green lines represent STRING interaction lines, and the red lines are generated based on the published literature (Zeng et al., 2022). **(B)** Representative enrichment results including Gene Ontology elements, pathways, and tissues corresponding to the PPI network. Terms are ranked based on the FDR value, and for each term, the number of corresponding genes is represented.

To understand the molecular cross-talk between MSCs and the OA microenvironment, the predicted secretome in day 14- and day 56-retrieved cells was investigated, identifying 157 and 278 predicted secreted genes, respectively (39 common elements) (Supplementary Figure S8). More secreted elements were found in the late retrieved MSCs, indicating that at this disease stage, with overt OA changes to cartilage and synovium, a stronger MSC–OA interaction may be present. Clusters were generated to mimic the cellular structure, and interaction networks were organized based on subcellular components (Zhu et al., 2015). In both time points, the highest number of elements were located in the membrane and extracellular components, in line with the top KEGG pathways corresponding to cytokine–cytokine interactions and cell adhesion molecules (Figures 4, 5). Enrichment analysis showed functional differences between the time points, indicating that the disease stage influences the nature of the cellular secretome. Based on enriched biological processes (BPs), on 14D, the network of proteins seemed to be mainly involved in ECM organization and collagen metabolism, whereas on 56D, these were concentrated on chemotaxis and immune responses. Similarly, these differences were further observed in the identified reactome pathways, where catabolic and anabolic processes correlated with the extracellular matrix and collagen were enhanced on 14D, while chemotactic receptors and their activation were upregulated on 56D. Mapping the elements to various tissue sources resulted in the identification of similarities between the retrieved cells and certain cell types. These followed the same time-point-related pattern since 14D-retrieved cells seem to be similar to synoviocytes and articular chondrocytes, whereas 56D-retrieved cells had similarities with granulocytes, neutrophils, and blood cells, potentially indicating a role in the immune response.

### 3.5 Validation of BRINP3 as a therapeutic target: protein expression and licensing effects on mesenchymal stromal cells

**FIGURE 6:**
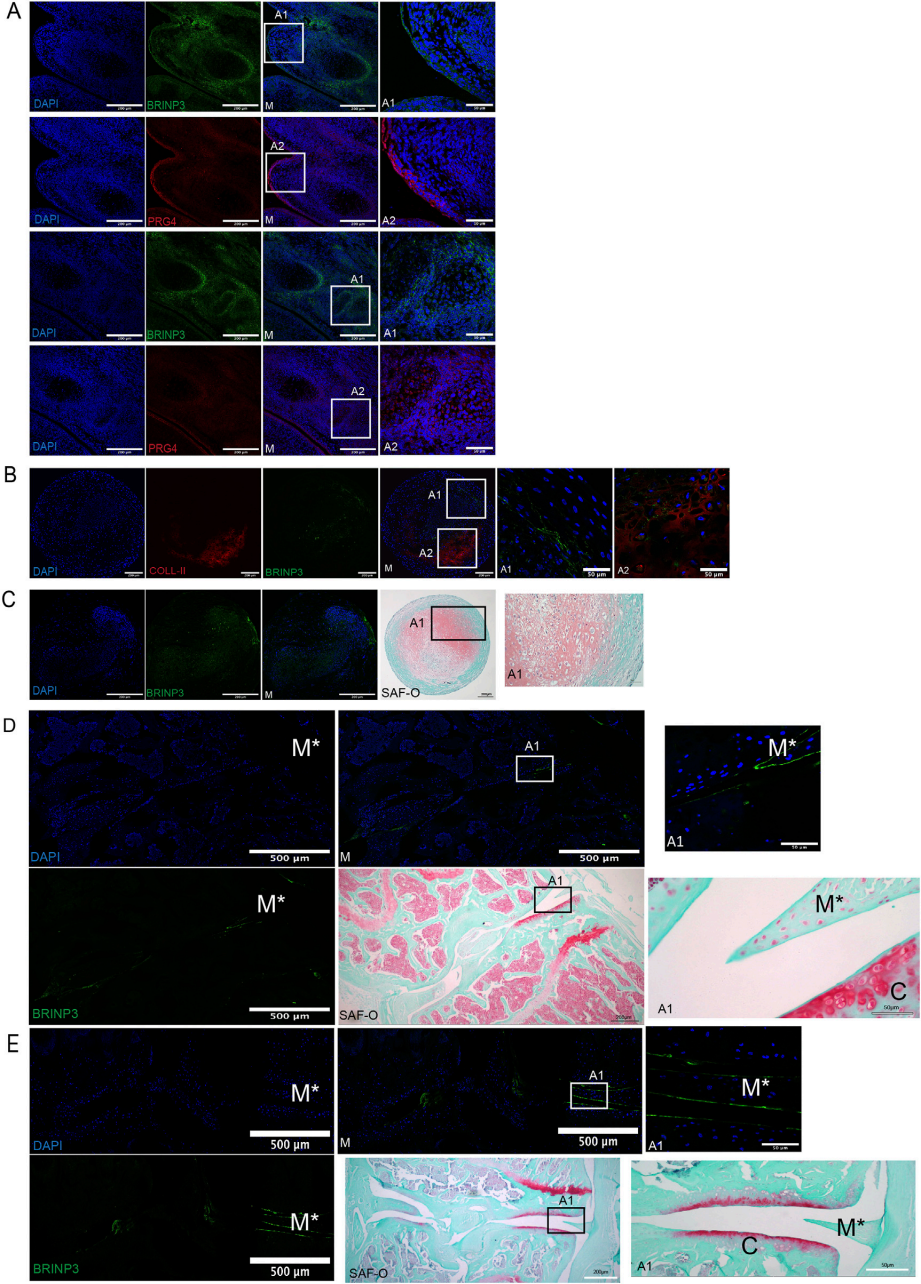
Validation of BRINP3 protein expression by immunofluorescence detection. **(A)** BRINP3 and PRG4 expression in developing digits of mouse embryos (E14–E15). **(B)** Co-expression of BRINP3 and collagen-II in the extracellular matrix in 28-day chondrogenically differentiated human BM-MSCs. **(C)** Expression of BRINP3 in the extra-cellular matrix and histological evidence of chondrogenic differentiation with Safranin-O staining in 28-day chondrogenically differentiated human ACPs (D/E). Immunofluorescent detection of BRINP3 in articular cartilage and meniscus in a murine CIOA **(D)** and DMM **(E)** model. For reference of anatomical structures, Safranin-staining of consecutive sections has been included for the CIOA and DMM models.

To further analyze the predicted secretome of retrieved cells as therapeutically licensed by the OA environment, we compared 14D and 56D datasets with the predicted secretome of control groups: *in vitro-*licensed MSCs with IL-6 alone or IL-6, MCP-1, and IFN-gamma combined (Supplementary Figure S9). *BRINP3*, identified as a novel predicted secreted gene expressed by BM-MSCs in all groups, was selected for further validation. The possible role of BRINP3 in synovial joint development in mouse embryo histological sections was assessed in tissues corresponding to gestational age E14–E15 (Martin, 1990), enabling the assessment of involvement in synovial joint cavitation and cartilage differentiation confirmed by the positive expression of PRG4 (Rhee et al., 2005). BRINP3 and PRG4 were detected at the superficial layer of the distal end of the digit and in the interdigital tissue. Both signals were detected in specific joint-forming locations comprised of cells undergoing active proliferation and differentiation, such as the remnant element of the joint interzone transiting toward cavitation. Chondrocytes located near the proximal and distal regions stained positive; however, signal intensity seemed to be reduced in cells, assuming a hypertrophic phenotype, and no signal was detected in the developing bone subchondral plate (Figure 6A). Given the premise that BRINP3 protein is highly conserved among vertebrates (Kawano et al., 2004) and to further assess its role in cartilage development, expressions of chondrogenically induced human MSCs and AC progenitor cells (ACPs) were assessed (Anderson et al., 2018). BRINP3 expression was identified in both cell types at D28 (Figures 6B, C), with higher magnification (×60) images revealing a diverse pattern of the BRINP3 signal. Expression in MSC pellets was identified in the cell cytoplasm and ECM as positive for collagen II staining, as well as in the external pellet layer, suggesting that MSCs under these conditions might synthesize and secrete BRINP3. ACPs also formed chondrogenic pellets, as confirmed by Safranin-O staining, containing fibroblast-like cells that generated a dense ECM, where the BRINP3 signal was detected in cells and the pericellular matrix surrounding the cells.

The expression of BRINP3 was further assessed in animal-induced OA models to examine its possible role in cartilage degradation. In murine CIOA and surgical destabilization of the medial meniscus (DMM) models (Farrell et al., 2016; Figures 6D, E), the BRINP3 signal was detected in a monolateral pattern on the meniscal and articular cartilage surface, corresponding to the healthy section of the joint. The lack of proteoglycans evidenced with Safranin-O staining was evidenced at the contralateral joint structures in both models, suggesting that BRINP3 expression might be lost during the pathophysiological process affecting the OA joint.

From a functional perspective, licensing with recombinant BRINP3 contributed to the maintenance of a normal fibroblast-like morphology in human MSCs. In terms of immunomodulatory effects, there was an absence of IDO secretion and increased gene expression of PTGS-2, hence potentially affecting the secretion of PGE2, a known modulator of anabolic and catabolic processes in OA (Goldring and Berenbaum, 2004). This finding suggests a possible role in the immunomodulation in OA (Supplementary Figure S10).

## 4 Discussion

The successful translation of MSC-based therapies from bench to bedside is still a persistent challenge in clinical practice. The missing link correlating mechanistic insights and clinical efficiency is an important barrier to the design of disease-relevant potency assays and effective clinical decision-making. To address the issue, we describe a strategy to characterize therapeutic mechanisms underpinning local MSC effects, whether delivered as a therapy to prevent the development of the disease post-joint injury or used to treat established OA. BM-MSCs were introduced *via* IA injection in a syngeneic murine CIOA model, mimicking autologous BM-MSC-based treatments, and surviving BM-MSCs were retrieved for analysis.

The results demonstrated that (1) the majority of cells do not survive 3-day post-delivery with impaired cell survival more evident in sham-injected mice, suggesting that the inflammatory milieu induces a survival stimulus; (2) retrieved BM-MSCs can secrete trophic factors capable of polarizing macrophages to an anti-inflammatory IL-10-producing phenotype; and (3) the transcription profile differs between cells retrieved in early and late-stage OA, indicating the important role of host environments in coordinating therapeutic outcomes.

This indicates that MSCs act *via* a hit-and-run mechanism characterized by short interactions with the inflammatory-diseased microenvironment, cell death, and lack of significant cell engraftment (Cheung et al., 2019) that does not, however, hinder their therapeutic efficacy (Murphy et al., 2003).

To assess the subsequent therapeutic effects, we investigated the immunomodulatory abilities of sham- and CIOA-retrieved BM-MSCs. Conditioned medium from retrieved cells could alternatively polarize *in vitro*-activated BMDMs, resulting in the lower surface expression of MHC-II and CD86 and increased expression of IL-10 known to be secreted by anti-inflammatory macrophages (Melief et al., 2013a; Melief et al., 2013b). This is in line with increasing evidence on immunomodulatory interactions between apoptotic MSCs and local microenvironments through the release of membrane particles and direct interaction with host monocytes and macrophages, resulting in the secretion of IL-10, TGF- β, IDO, and PGE2 (Antonio et al., 2017; Morrison et al., 2017; Masterson et al., 2019; Giacomini et al., 2023; Schrodt et al., 2023).

In this study, retrieved- and naïve *in vitro*-expanded BM-MSCs were similar in their morphology and cell surface marker expression, although transcriptomic analysis revealed differences associated with gene patterns and pathways involved in OA modulation and the chondrogenic phenotype. This confirms the hypothesis that upon transplantation, cell differentiation pathways can be activated or arrested, which could explain the observed differences in the retrieved cell data (Friedenstein et al., 1966). Our results imply that certain cell populations have an intrinsic enhanced ability to survive or that the inflammatory milieu induces transcriptional changes, promoting survival in responding cells. Differences in early- and late-CIOA-retrieved BM-MSCs reflect a direct cell response to OA disease status. Early stages are characterized by structural matrix-associated anabolic- and catabolic-driven changes; data revealed a distinct mapping profile related to the restoration of ECM organization and cell migration in early CIOA-retrieved BM-MSCs, a possible response to stimuli that accompany initial stages of joint synovitis with ECM degradation (Seol et al., 2014), aiming to resurface the joint and provide chondroprotection. Surviving cells in established OA, characterized by chronic inflammation and significant structural changes, were enriched for immune response regulation pathways known to counteract and modulate disease progression (Leijs et al., 2012). The inflammatory milieu may activate mechanistic cascades controlling cell death and differentiation, paracrine signaling, and immunomodulation, underlining the importance of injury and the local inflammatory status for successful cell treatments. The database of secretory gene expression generated represents a valuable catalog of the *in vivo* profile of BM-MSCs in the context of experimental osteoarthritis. However, future work needs to elucidate whether these mechanisms and associated pathways can be activated after cell transplantation in naturally occurring OA in both human and veterinary patients.

Although different expression profiles were observed between early- and late-retrieved BM-MSCs, progenitor cell markers active during limb development were characteristic of surviving BM-MSCs.

Differential expressions of genes with fundamental roles in synovial joint organogenesis were identified (PRG4 and GREM-1), suggesting the involvement of embryonic and fetal development pathways in surviving BM-MSCs. The potential therapeutic cell capacity can be correlated with the ability of putative chondroprogenitor PRG4+ cells to restore the AC surface in an *in vivo* cartilage wound model (Massengale et al., 2023). These cells express a pattern of unique chondrocyte-like markers, distinguishing naïve MSCs from mature chondrocytes, similar to what we identified in our study.

The BRINP3 signal was observed for the first time on the surface of AC and meniscus in a mouse OA model. BRINP3/FAM5C is diversely methylated in human OA being hypomethylated in cartilage (Fernández-Tajes et al., 2014) and hypermethylated in subchondral bone in intermediate-late disease phases (Zhang et al., 2016). However, it is unknown whether its methylation status contributes to OA onset and progression or is merely a consequence of joint tissue changes in response to synovial inflammation, potentially driven by the upregulation of pro-inflammatory cytokines such as TNF-α, IL-6, IL-1β, and LPS (Sato et al., 2014). Here, BRINP3 was tested as a licensing factor in human BM-MSCs and showed the ability to regulate the gene expression of key inflammatory mediators in OA, resulting in the upregulation of PTGS-2, associated with MSC-mediated immunosuppression (Paradiso et al., 2022) and the downregulation of TSG-6, one of the most highly upregulated genes in OA associated with cartilage degradation (Chou et al., 2018). Further studies are needed to fully elucidate the effect of BRINP3 and, in addition, the cumulative effect of multiple cytokines and immune cell populations present in the OA synovial fluid.

BRINP3 is involved in bat short digit development (Wang et al., 2014), and it is also expressed in human embryonic stem cells and derived chondrogenic progenitors (Griffiths et al., 2020). The current literature, along with this study’s findings, indicate that BRINP3, involved in human limb development, could be a novel factor expressed by surviving BM-MSCs in response to the OA inflammatory milieu. Molecular analysis data of retrieved cells also contribute to the premise that (1) BM-MSCs exposed to the joint inflammatory milieu may assume a cartilage-derived progenitor/stem cell-like phenotype or (2) a specific subpopulation of adult MSCs, known to express transcripts of embryonic cell types, survive as progenitor-like cells with enhanced capacity to engraft, survive, and mediate tissue repair and/or joint protection.

The limitations to our study include the number of pooled retrieved cells because the size of the mouse joint and larger animal models would be able to yield a higher number of cells, allowing a more comprehensive validation of the predicted secretome. Performing an additional analysis of the *in vivo* apoptotic profile of IA-injected BM-MSCs would enable the elucidation of the mechanisms involved in cell death and correlate them with desired therapeutic outcomes.

Our data highlight species-related differences in skeletal regulatory pathways between mice and humans, which must be considered for the translational value of this work. The database of secretory gene expression generated represents a valuable catalog of the *in vivo* profile of BM-MSCs in the context of experimental osteoarthritis. Furthermore, the relevance of this secretory gene expression profile needs to be considered for the design of potency assays, the evaluation of quality control of MSC or MSC-derived therapeutic products, and the development of new clinical strategies for OA. Addressing these gaps will be beneficial for understanding and optimizing clinical applications of the cells and defining pharmacokinetic and pharmacodynamic parameters necessary for any medicinal product used in the clinical setting.

## Data Availability

The raw data supporting the conclusions of this article will be made available by the authors, without undue reservation.
