# Supplementary material for: Identification of disease-stage therapeutic responses of mesenchymal stromal cells retrieved from murine osteoarthritic joints
Source: Front Cell Dev Biol. 2025 Mar 26;13:1521437. doi: 10.3389/fcell.2025.1521437 (PMC11980424; doi:10.3389/fcell.2025.1521437)
Supplement: Supplementary file 1 [file DataSheet2.docx]

**SUPPLEMENTARY MATERIALS AND METHODS**

- 1. **Cryoimaging**

For CryoViz^TM^ Imaging murine wild-type BM-MSCs were labelled with quantum dots (QTracker^®^ 625 Cell Labelling Kit, Invitrogen) following manufacturer’s instructions. Briefly, 1x10^6^ cells were incubated with 20nM QDot solution on a shaker (80rpm) for 1 hour at 37°C, washed twice in cell culture medium and resuspended in PBS. 200,000 cells were injected one week after CIOA induction. Mice were culled immediately and at 72 hours post-injection. Hind limbs were dissected and mounted in OCT (BioInvision Inc., Cleveland, OH, USA). Sectioning of the samples and imaging analysis was performed using an automated CryoViz^TM^ imaging system (BioInvision, at Maynooth University) (1).

- 1. **Immune modulation assays**
     1. Suppression of T cell proliferation

Spleens were obtained from 8-10-week-old C57BL/6 mice and single cell suspensions were generated by filtering the organ through a 22 μm cell strainer. Cells were then centrifuged at 800 x g for 5 min and supernatant was discarded. To eliminate red blood cells, the pellet was resuspended in 1 ml of ACK lysis buffer (Sigma-Aldrich) and incubated for 5 min at 4 °C in the dark. The lysis buffer was then neutralised with PBS, and re-suspended at the concentration of 10x10^6^cells/ml. Cell Trace Violet (CTV, Invitrogen, Thermo Fisher Scientific) was added at the concentration of 1 μl/ml and incubated for 20 min at RT in the dark. Cells to be activated were re-suspended at the concentration of 1x10^6^ cells/ml and the soluble antibodies anti-CD3e (eBiosciences 16-0031-85) and anti-CD28 (eBiosciences 16-0281-85) were added. 1x10^5^ cells were seeded in 100 μl in each well of a U-bottom 96-well plate. MSCs from the control, SHAM and CIOA group were seeded over the lymphocytes at different ratios (1:5 and 1:2) in 100 μl of MSC medium. After 3 days splenocytes were collected and stained for flow cytometric analysis using the antibodies listed in Supplementary Table S3. Prior to analysis, 1 μl of viability dye DRAQ7 was added to each well.

- - 1. Inhibition of macrophage activation

Macrophages were derived by differentiation of whole BM with M-CSF derived from L929 (ATCC no. CCL-1) supernatant, as previously described(Zhang et al., 2008). Briefly, BM cells were obtained from long bones of C57BL/6 mice and plated in 6-well plates at 3x10^5^/well with BMDM differentiation medium (RPMI-1640 supplemented with 15% L929 supernatant, 10% FBS, 2mM L-glutamine, 0.1% non-essential amino acids, 1mM sodium pyruvate, 50µM 2-β-mercaptoethanol and 1% P/S), with medium changes performed every 2-3 days. Differentiation was confirmed at day 6 by flow cytometry using anti-CD11b (BD Pharmingen), anti-CD45.2 and anti-F4/80 (BioLegend) with isotype controls differentiating non-specific background signal (Supplementary Table S4). Differentiated BMDMs were primed with IFN-γ (Peprotech, 20ng/ml) and seeded at 2x10^5^/well in 24-well plates. The following day, cells were activated with LPS (Sigma-Aldrich, 10 ng/ml) and treated with 50% MSC-CM. After 4h, medium was collected for ELISA and NO quantification and cells were harvested and stained for CD206, CD86, F4/80 and I-A/I-E (MHC-II) (BioLegend) (Supplementary Table S4). Details on

- 1. **Identification of the in vivo inflammatory profile of the CIOA model**

All procedures were approved by the Animal Care and Research Ethics Committee of the National University of Ireland, Galway and licensed by the Health Products Regulatory Authority (HPRA), Ireland. Eight-week-old male C57BL/6 mice were supplied by Charles River Laboratories, Ballina, Ireland. Six animals were assigned to each time point, i.e. Day 0, 7, 14, 21, 28, 49 and 56, three as sham (untreated) and three as collagenase-induced OA (CIOA) (treated).

To induce OA, 1 U of highly purified bacterial type VII collagenase (Sigma-Aldrich) in 6 μl of physiological saline (vehicle) was injected into the knee joint of mice twice, two days apart (totaling 2 U). Animals were anaesthetised using isoflurane (5 to 1 – 2%) and the knees shaved and aseptically prepared prior to collagenase injection. At the given time points, i.e. Day 0, 7, 14, 21, 28, 49 and 56 after the induction of OA, animals were euthanised. The synovial fluid was harvested as follows: following removal of all excess tissue from the joint, an incision was created in the synovial capsule, thereby exposing the joint. The exposed joint was then placed in 50 µL D-PBS in a 96-well flat-bottomed plate (Sarstedt, Wexford, Ireland) and rocked at 4^o^C for 1 hour. Dissected joints were individually placed in 50 μl PBS for 2 consecutive periods of 15 min with fluid collected after a short spin after each incubation period before pooling of approximately 120 μl. Washouts were pooled per animal, centrifuged at 1500 rpm for ten minutes and stored at -80^o^C for Bioplex Analysis using the Mouse Magnetic Luminex Kit (L122149, LXSAMSM-23, R&D Systems) following the manufacturers’ instructions. Dissected joints were individually placed in 50 μl PBS for 2 consecutive periods of 15 min with fluid collected after a short spin after each incubation period before pooling of approximately 120 μl.

Popliteal lymph nodes, inguinal lymph nodes and spleen were harvested and analysed for T-cell and Macrophage markers by flow cytometry. Following removal of popliteal and inguinal lymph nodes and spleen from the C57BL/6 mice, single cell suspensions were obtained from each animal. Popliteal lymph nodes from three animals were pooled due to low cell numbers. The inguinal lymph nodes and spleen were kept as 1 sample per animal. Cell suspensions (1 x 10^5^ cells/well) were stained in triplicate in a 96 well v-bottomed plate (Sarstedt, Wexford, Ireland) according to optimised conditions for cell surface expression of CD4 (anti-mouse CD4, Biolegend 100412, Clone GK1.5), CD8 (anti-mouse CD8, Biolegend 100712, Clone 53-6.7), CD25 (anti-mouse CD25, Biolegend 102042, Clone PC61), and CD3 (anti-mouse CD3, Biologend 100204, Clone 17A2). Cell suspensions were stained according to optimised conditions for cell surface expression of CD11b (anti-mouse CD11b, Biolegend 101226, Clone M1/70), MHC1 (anti-mouse MHCI, Biolegend 111512, Clone KH95), MHCII (anti-mouse MHCII, Biolgened 116410, Clone AF6-120.1), CD86 (anti-mouse CD86, Biolegend 105040, Clone GL-1) to characterise macrophage subsets. All samples were stained using anti-mouse Sytox Blue (Brilliant Violet 421, Life Technologies, Dublin, Ireland) to discriminate live from dead cells. Samples were analysed on a FACS Canto cytometer (BD Biosciences).

- 1. **Licensing of murine GFP+ BM-MSCs**

GFP-MSCs isolated from bone marrow and expanded from C57BL/6-Tg(UBC-GFP)30Scha/J mice were seeded in triplicate at a density of 4 x 10^4^ cells/well in a 12-well plate in MSC medium (αMEM with Glutamax, 10% FBS, 1% penicillin/streptomycin) and incubated at 37°C and 5% CO_2_ in a humidified incubator. Following attachment overnight, cells were treated with 50 ng/ml of either IL-6 or the combination of MCP-1, IL-6 and IFN-γ, each at 50 ng/ml (Peprotech, UK) or remained untreated. Cells were harvested for analysis at 72 hours post treatment for RNA sequencing carried out as described for the retrieved MSCs.

- 1. **Bioinformatics Analyses**

FASTQ files containing sequenced reads were quality checked using FASTQC v.011.5(Andrews, 2010), and quality and adapter trimming performed using Trimmomatic v.0.36(Bolger et al., 2014). Salmon v0.8.1, which uses a quasi-mapping algorithm to provide expression estimates of RNA sequencing data, was used for transcript mapping and quantification of trimmed reads(Patro et al., 2017). An index of the relevant transcriptome was built using the Mus Musculus GRCm38 transcriptome from ENSEMBL (<ftp://ftp.ensembl.org/pub/release-96/fasta/mus_musculus/cdna/>) with the paired end data serving as input files for transcript quantification by Salmon. Parameters were set to automatically detect the library type and correct for GC-bias. Mapping rates were assessed for quality of mapping and contamination. Samples with exceedingly low mapping rates were not considered for downstream analysis. Output quantification files produced by Salmon were imported into R (v3.5.1) and processed by the Bioconductor package(Soneson et al., 2015) to obtain matrices of gene-level count data. Independent filtering of low abundance reads was performed in R v3.5.1; counts of <50 across samples were removed from data to filter out noise and improve sensitivity. For differential gene expression, CIOA samples were compared to their corresponding sham controls.

As pooled biological replicated data was used, differential gene expression was performed using GFOLD v1.1.4. GFOLD (generalised fold change), which performs size factor normalisation to account for differences in sequencing depth and derives a generalised log fold change based on the posterior distribution of the raw log fold change(Feng et al., 2012).

- 1. **Analyses of Differentially Expressed Genes**

Differentially expressed genes were considered to exceed an absolute log 2fold change of 1. Expression data and predicted upstream regulators were analyzed using Ingenuity Pathway Analysis (IPA; QIAGEN Inc.). The predicted secretome analysis was performed on differentially expressed genes using Proteinside (Kaspric et al., 2015). Gene set enrichment to obtain Biological Processes, Molecular Functions and Cellular Components were performed using EnrichR(Kuleshov et al., 2016). Heatmaps were generated using Morpheus(Morpheus, n.d.) and downstream analyses were enabled in R using graphics v3.5.1 and ggplot2 v3.1.1.

- 1. **Cell proliferation and clonogenic potential of naïve and retrieved MSCs**

To assess proliferation, cells were seeded in a 6-well plate at 1x10^5^ cells/well and passaged every 24h. Clonogenic potential was assessed by seeding 1000/500/ 250 cells in a 6-well plate in MSC medium for one week. Cells were fixed with methanol and stained with 0.5% crystal violet solution (Sigma-Aldrich). Clusters of at least 20 cells were counted and clonogenicity calculated using: *CFU-F = (CFU/N_s_)×100*, where *CFU-F* is the colony forming efficiency, *CFU* the number of colonies counted and *N_s_* the number of seeded cells.

- 1. **BRINP3 as licensing factor for human BM-MSCs**

Human BM-MSCs (n=3 donors) were cultured in 25 cm^2^ culture flasks at a density of 5000 cells/cm^2^ in normoxic conditions (37°C, 5% CO_2_, 95% humidity) in culture medium composed of 90% α-MEM (Gibco), 1% penicillin/streptomycin combination (p/s, Gibco), 10% fetal bovine serum (FBS, Sigma) and 1 ng/mL of fibroblast growth factor 2 (FGF2). For licensing experiments, hBM-MSCs were plated as described above and 24h post-seeding, licensing media containing 10 ng/ml, 25 ng/ml, 50 ng/ml, and 100 ng/ml of recombinant human FAM5C/BRINP3 (ab166266, Abcam) was added individually for 72h. To evaluate the production of Indoleamine 2,3-Dioxygenase 1 (IDO1), µl of culture supernatants were treated with 50 µl of 30% trichloroacetic acid (TCA; Sigma Aldrich), incubated at 50°C for 30 min. 75 µl from each well were transferred to a new 96-well plate followed by addition of an equal volume of Ehrlich reagent (Sigma Aldrich; 1% p-dimethylbenzaldehyde in glacial acetic acid). OD was measured at λ =492 nm in VICTORX™ (Perkin Elmer). The amount of L-kyn was determined using a standard curve of L-kyn (0–200 µM).

To analyze the gene expression of anti-inflammatory mediators in licensed MSCs, cells were detached with 0.25% 1X Trypsin-1mM Ethylene diamine tetra-acetic acid (Trypsin-EDTA; Gibco) for 5 min at room temperature and stored in lysis buffer at -80°C. Total RNA was isolated from frozen BM pellets using the RNeasy Mini Kit (Qiagen) following the manufacturers’ instruction and nucleic acid content was determined using the NanoDrop ND1000 Spectrophotometer (ThermoFisher Scientific) at 260 and 280nm. First strand cDNA synthesis was performed using the RNA the Superscript® VILO™ cDNA Synthesis kit (Thermo Fisher Scientific) using a LifeTouch thermal cycler (BIOER) following the manufacturers’ instructions.

qRT-PCR was performed in MicroAmp® Fast 96-well Reaction Plate (Thermo Fisher Scientific) using the QuantiNova SYBR Green PCR Kit (Qiagen) or the Fast SYBR™ Green Master Mix (Thermo Fisher Scientific). Each combination of primer and sample was repeated with three replicates. For each combination No Template Controls (NTC) in three replicates were also prepared. PCR kit). The plates were sealed tightly with Optical Adhesive covers (Thermo Fisher Scientific). qRT-PCR was performed in a StepOnePlus™ Real-Time PCR System (Thermo Fisher Scientific). The following primers purchased from IDT were used at a concentration of 700nM: actin beta (ACTB) (Hs.PT.39a.22214847) as housekeeping gene, TNF-alpha induced protein (TNFAIPC/TSG-6) (Hs.PT.58.3630286), interleukin 10 (IL-10), (Hs.PT.58.2807216), prostaglandin-endoperoxide synthase 2 (PTGS2) (Hs.PT.58.77266), IDO1 (Hs.PT58.924731) and interleukin 6 (IL-6) (Hs.PT.5840226675).

- 1. **Cytoscape (v3.9.1) diagrams**

Protein-protein interaction (PPI) network was created using STRINGapp (ver.1.71), where 2 networks, day14 and day56 (secreted proteins both up and down regulated). Clustering and annotation were carried out using Autoannotate (Ver 1.3.5) and annotation was based on using localisation results column imported from CellWhere (column labelled “Cellwhere Localization) (<https://www.sys-myo.com/cellwhere/>). Nodes that displayed gene names as “ENSMUSP***********” were changed to symbols from “query term column”.

Functional enrichment was carried out on webtool for both networks (due to lack of option in uploading custom gene background for Cytoscape that would incur high memory usage if full list was added as network). Custom background for each network were based on all the genes detected in each comparison (d14 Control vs d14 OA and d56 control vs d56 OA).

**1.10. CellWhere analyses**

Gene lists were uploaded in batches of 50 at a time (due to webtool constraints) for potential secretome proteins. Settings were to mouse genome, input identifier was set as “Gene symbol”, localisation resources as “Uniprot” and GO”, and localisation displayed as annotation only, Mentha interactor settings were ignored. Each batch had data saved by copying and pasting to csv/excel files; genes that could be identified using Uniprot after failing first pass using symbol were highlighted in orange. Any genes which did not have a corresponding localisation result were marked as unknown in results due to lack of identification in either gene symbol or UniProt accession number have been listed and highlighted in yellow in Supplementary Table S12. Those that had no symbol but could be assessed with UniProt were marked in orange in Excel(Zhu et al., 2015).

**1.11. Generation of Heatmap of TPMS from Venn diagram**

Tximport was used to obtain TPM values from samples. Heatmap was used to create the heatmap. R version 4.2 was the programming language used to produce figures. Included are names which did not match between ensemble annotation and symbols from expression data; these were double-checked with mouse ensemble database and are found in Table S12. Code is provided to recreate the figure.

- 1. **. Safranin-O staining**

Selected slides were stained as follows: 100% xylene for 5min, 100% xylene for 5min, 100% IMS for 2min, 100% IMS for 2min, 95% IMS for 1min, 70% IMS for 1min, dH2O 1min, 0.02% Fast Green FCF (w/v) (Sigma) for 4min, 1% acetic acid (Sigma) for 3s, 0.1% Safranin-O (Sigma) for 6min, 95% IMS for 1min, 100% IMS for 2min, 100% IMS for 2min, 100% xylene for 2min, 100% xylene for 2min. The slides were mounted with the distyrene plasticizer xylene (DPX; Sigma), cover-slipped and left to air dry in the fume hood.

**References:**

Andrews (2010). FASTQC. Available at: https://www.bioinformatics.babraham.ac.uk/projects/fastqc/

Bolger, A. M., Lohse, M., and Usadel, B. (2014). Trimmomatic: A flexible trimmer for Illumina sequence data. *Bioinformatics* 30, 2114–2120. doi: 10.1093/bioinformatics/btu170

Carty, F., Corbett, J. M., Cunha, J. P. M. C. M., Reading, J. L., Tree, T. I. M., Ting, A. E., et al. (2018). Multipotent adult progenitor cells suppress T cell activation in in vivo models of homeostatic proliferation in a prostaglandin E2-dependent manner. *Front Immunol* 9, 1–14. doi: 10.3389/fimmu.2018.00645

Feng, J., Meyer, C. A., Wang, Q., Liu, J. S., Liu, X. S., and Zhang, Y. (2012). GFOLD: A generalized fold change for ranking differentially expressed genes from RNA-seq data. *Bioinformatics* 28, 2782–2788. doi: 10.1093/bioinformatics/bts515

Kaspric, N., Picard, B., Reichstadt, M., Tournayre, J., and Bonnet, M. (2015). ProteINSIDE to easily investigate proteomics data from ruminants: Application to mine proteome of adipose and muscle tissues in bovine foetuses. *PLoS One* 10, 1–24. doi: 10.1371/journal.pone.0128086

Kuleshov, M. V., Jones, M. R., Rouillard, A. D., Fernandez, N. F., Duan, Q., Wang, Z., et al. (2016). Enrichr: a comprehensive gene set enrichment analysis web server 2016 update. *Nucleic Acids Res* 44, W90–W97. doi: 10.1093/nar/gkw377

Morpheus (n.d.). Available at: https://software.broadinstitute.org/morpheus (Accessed December 29, 2020).

Patro, R., Duggal, G., Love, M. I., Irizarry, R. A., and Kingsford, C. (2017). Salmon provides fast and bias-aware quantification of transcript expression. *Nat Methods* 14, 417–419. doi: 10.1038/nmeth.4197

Soneson, C., Love, M. I., and Robinson, M. D. (2015). Differential analyses for RNA-seq: transcript-level estimates improve gene-level inferences. *F1000Res* 4, 1521. doi: 10.12688/f1000research.7563.1

Zhang, X., Goncalves, R., and Mosser, D. M. (2008). The isolation and characterization of murine macrophages. *Curr Protoc Immunol*, 1–14. doi: 10.1002/0471142735.im1401s83

Zhu, L., Malatras, A., Thorley, M., Aghoghogbe, I., Mer, A., Duguez, S., et al. (2015). CellWhere: Graphical display of interaction networks organized on subcellular localizations. *Nucleic Acids Res* 43, W571–W575. doi: 10.1093/nar/gkv354
