# Supplementary material for: Identification of disease-stage therapeutic responses of mesenchymal stromal cells retrieved from murine osteoarthritic joints
Source: Front Cell Dev Biol. 2025 Mar 26;13:1521437. doi: 10.3389/fcell.2025.1521437 (PMC11980424; doi:10.3389/fcell.2025.1521437)
Supplement: Supplementary file 2 [file DataSheet1.docx]

**SUPPLEMENTARY INFORMATION**


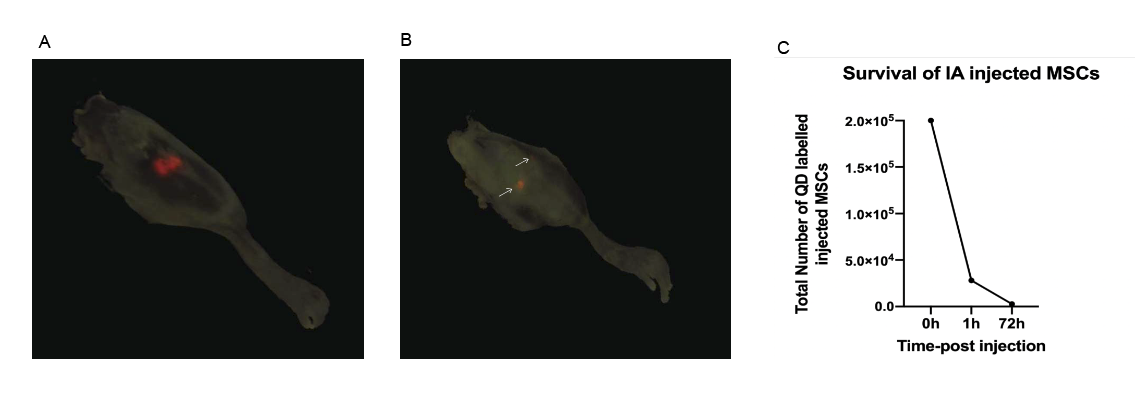


**Fig. S1. CryoViz Imaging of murine hind limbs.** IA-delivery of QDot labelled wild-type murine MSCs showed a positive signal cluster at the injection point at 1 hour post injection (A) and localized minor signals at 72 hours post injection (B) showing a rapid decline in cell survival (C).


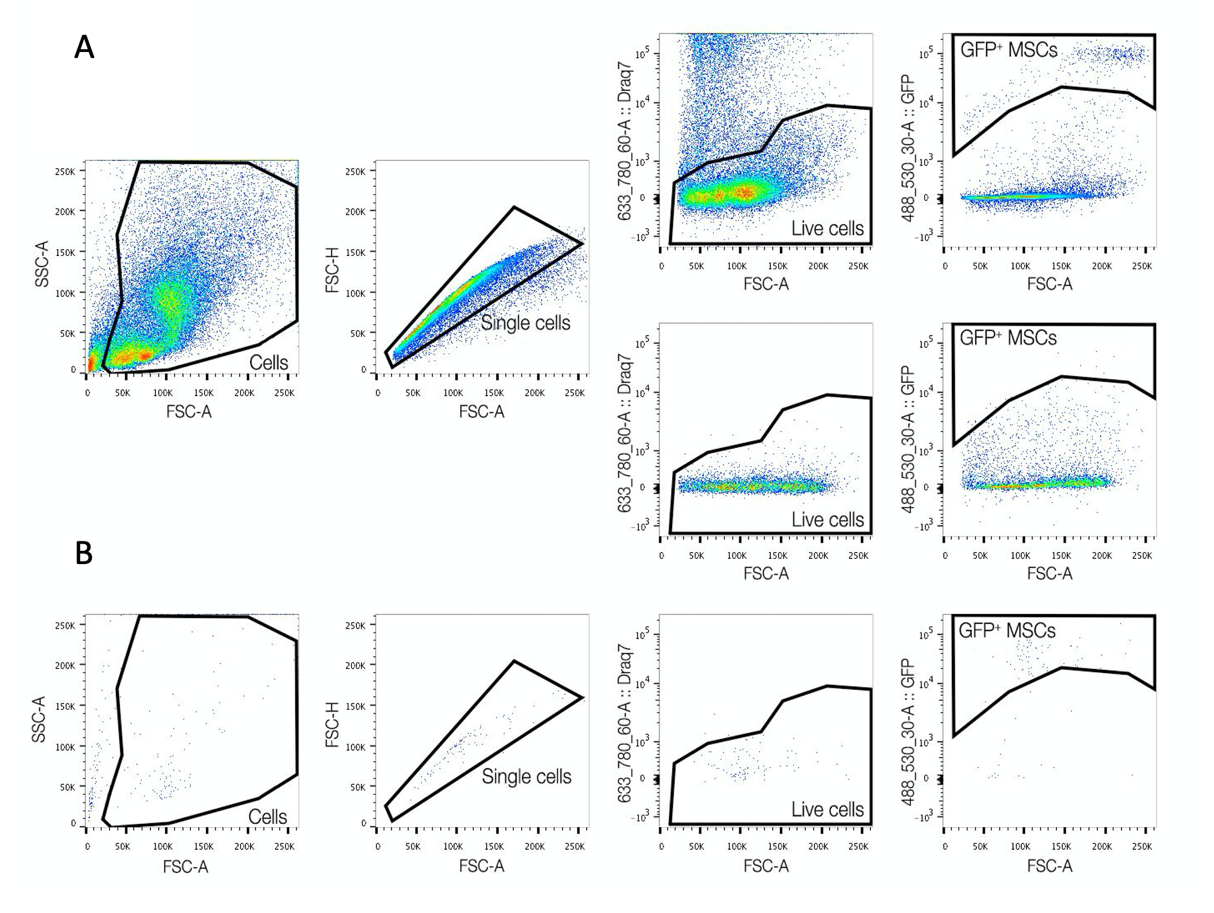


**Fig. S2. Cell sorting of IA-administered GFP+ MSCs**. Cell debris was gated out from the scatter plot. Doublets were excluded and live cells selected as DRAQ7 positive. GFP+ MSCs were identified using a negative control and the gate was used for cell sorting (A). Sorted cells were analysed for purity using the same gating strategy (B).

| Receptor | Isotype | Clone | Fluoro-chrome | Supplier |
| --- | --- | --- | --- | --- |
| CD105 | Rat IgG2a, κ | MJ7/18 | APC | BioLegend (120413) |
| CD106 | Rat IgG2a, κ | 429 (MVCAM.A) | PE | BioLegend (105713) |
| CD140b | Rat IgG2a, κ | APB5 | APC | eBioscience (17-1402-80) |
| CD146 | Rat IgG2a | ME-9F1 | PE | BioLegend (134703) |
| CD29 | Armenian hamster IgG | eBioHMb1-1 (HMb1-1) | APC | eBioscience (17-0291-80) |
| CD44 | Rat IgG2b, κ | IM7 | PE | BioLegend (103007) |
| CD90.2 | Rat IgG2a, κ | 53-2.1 | PE | BD Pharmingen (553005) |
| Ly-6A/E (Sca-1) | Rat IgG2a, κ | E13-161.7 | APC | BioLegend (122511) |
| CD11b | Rat IgG2b, κ | M1/70 | PE | BD Pharmingen (557397) |
| CD45 | Rat IgG2b, κ | 30-F11 | PE | BD Pharmingen (553081) |

**Table S1. Antibodies for cell surface marker characterization**

| Experimental replicate | Joint condition | Yield | % Injected | % Purity |
| --- | --- | --- | --- | --- |
| Retrieval 1 | SHAM | 952 | 0.040 | / |
|  | CIOA | 7964 | 0.332 | / |
| Retrieval 2 | SHAM | 331 | 0.014 | 77.8% |
|  | CIOA | 634 | 0.026 | 88.9% |
| Retrieval 3 | SHAM | 251 | 0.010 | 69.2% |
|  | CIOA | 1067 | 0.044 | 90 % |

**Table S2: Cell sorting of retrieved MSCs from SHAM and CIOA murine joints**. MSCs were consistently retrieved in higher number from CIOA vs SHAM joints. Purity of the retrieved cell populations was analyzed for retrieval experiments 2 and 3 showing elevated values in SHAM (69-77.8%) and CIOA (88.9-90%) joints.


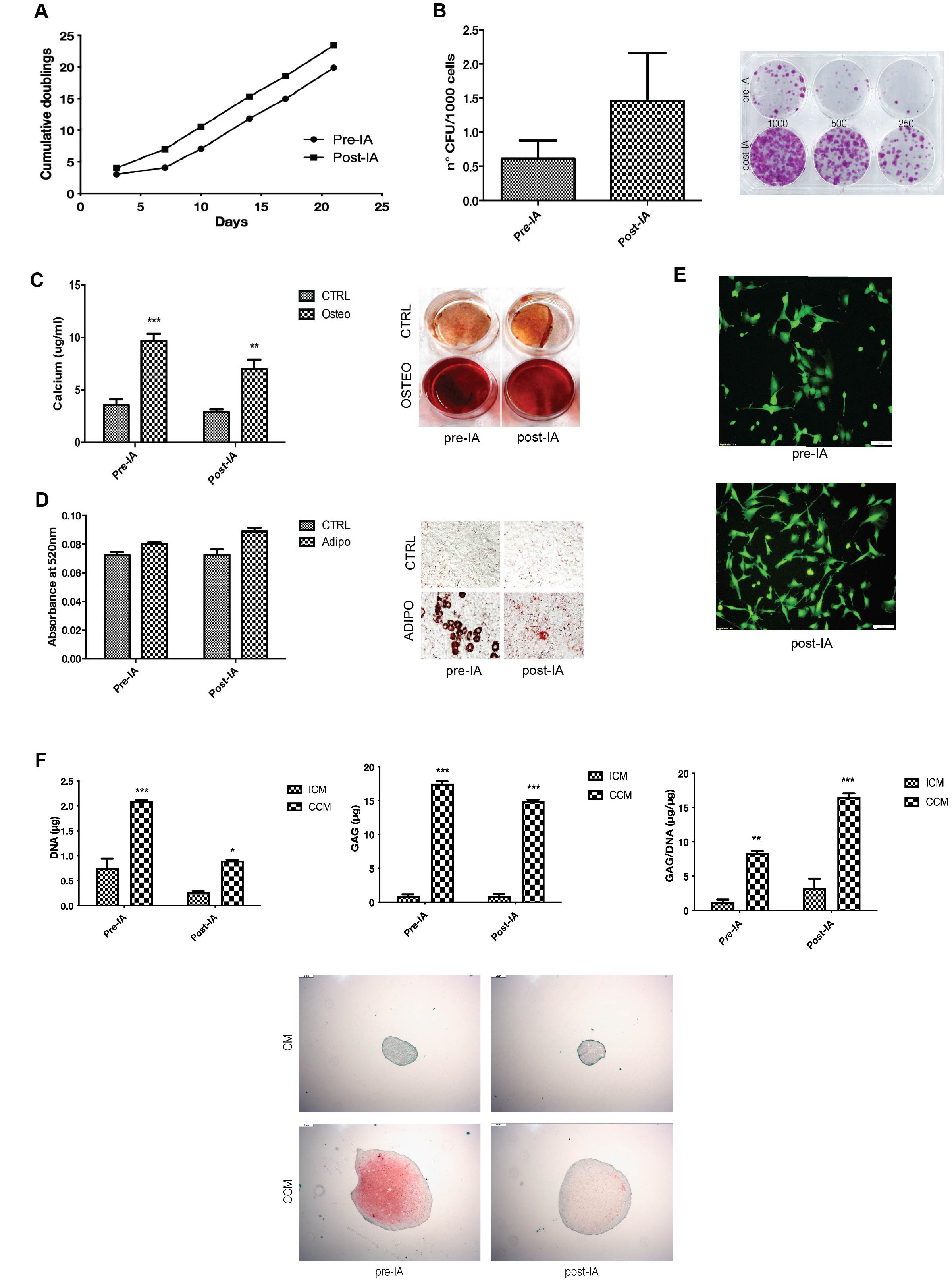


**Fig. S3. Characterisation of retrieved MSCs.** A: Proliferation rates of MSCs before (pre-IA) and after (post-IA) *in vivo* administration and FACS sorting (right) n=1. B: Clonogenicity of MSCs before (pre-IA) and after (post-IA) *in vivo* administration and FACS sorting (Mean±SEM, n=1, 3 technical replicates) C: Osteogenic differentiation of retrieved MSCs. Alizarin Red Staining (A) and calcium quantification (B) of MSC monolayers in osteogenic differentiation medium before (pre-IA) and after (post-IA) in vivo administration and FACS-sorting. Mean±SEM (n=1, 3 technical replicates). D: Adipogenic differentiation of retrieved MSCs. Oil red-O staining (A) and semi-quantification (B) of MSC monolayers in adipogenic differentiation medium, before (pre-IA) and after (post-IA) in vivo administration and FACS-sorting. Mean±SEM (n=1, 3 technical replicates) E: Representative inverted microscopy images of cultured MSCs before (pre-IA) and after (post-IA) *in vivo* administration and FACS-sorting. Scale bar = 100µm. F: Chondrogenic differentiation of retrieved MSCs. Safranin-O (red) and Fast Green (green) staining of chondrogenically-differentiated pellets (A). Incomplete Chondrogenic Medum (ICM), Complete Chondrogenic Medium (CCM). Quantification of glycosaminoglycans (GAG) (B), DNA (C) and GAG/DNA ratio (D) for chondrogenic-differentiated pellets of MSCs, before (pre-IA) and after (post-IA) *in vivo* administration and FACS sorting.

| Receptor | Fluorochrome | Clone | Supplier |
| --- | --- | --- | --- |
| **CD4** | PerCP/Cy5.5 | RM4-5 | BioLegend (100540) |
| **CD8** | APC | 53-6.7 | BioLegend (100712) |
| **CD25** | PE | PC61 | BioLegend (102008) |

**Table S3. Antibodies used in T cell co-culture analysis**

| Receptor | Isotype | Fluoro-chrome | Clone | Supplier |
| --- | --- | --- | --- | --- |
| **CD11b** | Rat IgG2b, κ | PE | M1/70 | BD Pharmingen (557397) |
| **Isotype Ctrl** | Rat IgG2b, κ | PE | A95-1 | BD Pharmingen (553989) |
| **CD45.2** | Mouse IgG2a, κ | APC | 104 | BioLegend (109813) |
| **Isotype Ctrl** | Mouse IgG2a, κ | APC | MOPC-173 | BioLegend (400221) |
| **F4/80** | Rat IgG2a, κ | PE/Cy7 | BM8 | BioLegend (123113) |
| **Isotype Ctrl** | Rat IgG2a, κ | PE/Cy7 | RTK2758 | BioLegend (400521) |
| **CD206** | Rat IgG2a, κ | Brilliant Violet 421 | C068C2 | BioLegend (141717) |
| **CD86** | Rat IgG2a, κ | Brilliant Violet 510 | GL-1 | BioLegend (105039) |
| **F4/80** | Rat IgG2a, κ | APC | BM8 | BioLegend (123115) |
| **I-A/I-E (MHC-II)** | Rat IgG2b, κ | PE | M5/114.15.2 | BioLegend (107607) |

**Table S4. Antibodies used for macrophage characterization**

**
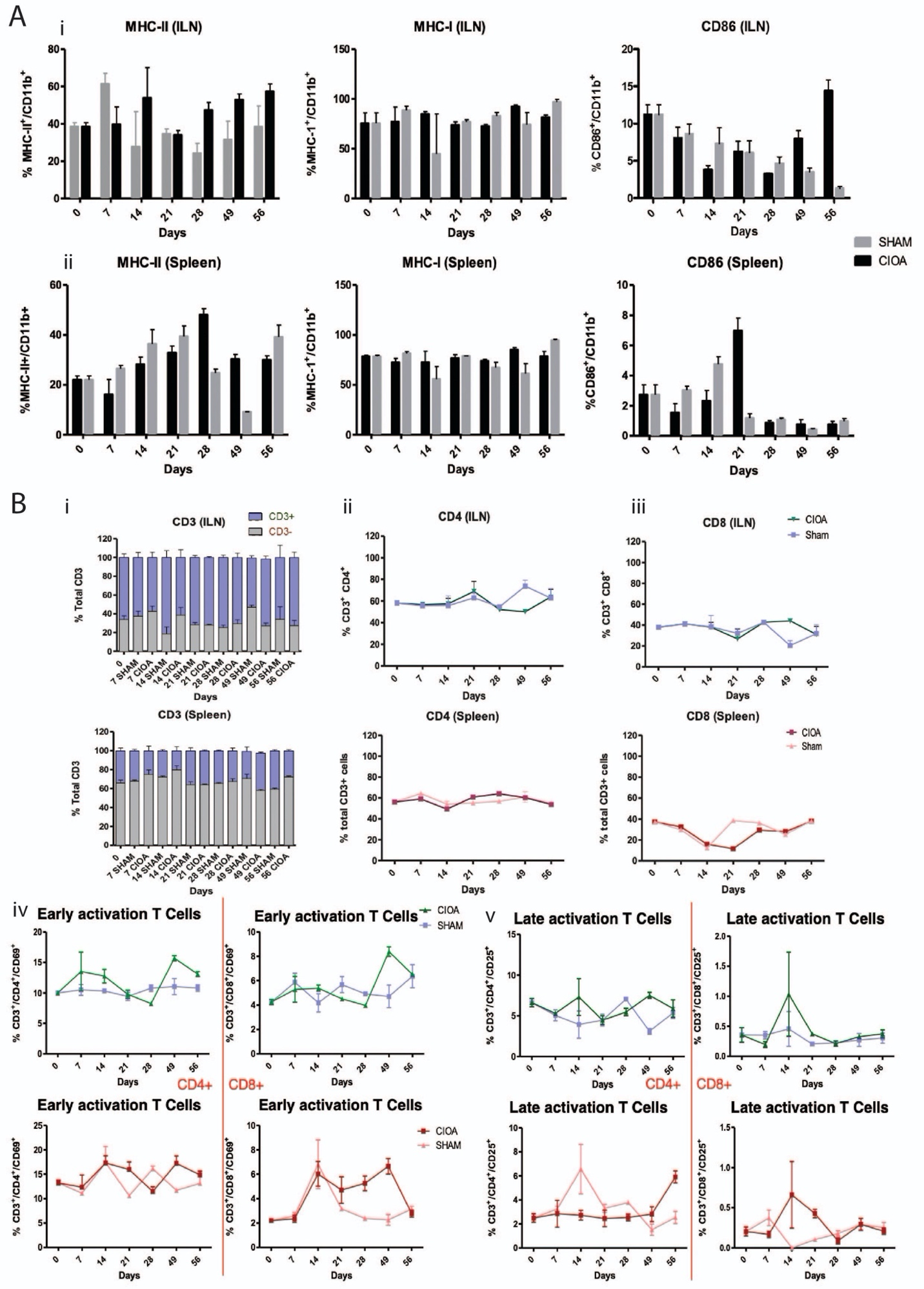
**

**Fig. S4. Identification of the *in vivo* immunological profile in a collagenase induced OA.** (A) Macrophage populations identified in the inguinal lymph nodes (i) and Spleen (ii) of Sham and CIOA groups over 56 days following CIOA. The % of MHC-II^+^/CD11b^+^ cells increased early (D14) in ILN and later (D28) in Spleen in CIOA versus Sham, while % of MHC-I^+^/CD11b^+^ cells increased in late OA (D49) in the Spleen. An increase in the % of CD86^+^/CD11b^+^ cells was also observed late (D56) in ILN and early (D21) in Spleen. (B) T cell populations found within the inguinal lymph nodes and Spleen of Sham and CIOA groups over 56 days following CIOA: CD3^+^ (i), CD3^+^/CD4^+^ (ii), CD3^+^/CD8^+^ (iii). Activation of T cells was analysed via measurement of CD69^+^ (iv) and CD25^+^ (v) populations.

| Cytokines | |
| --- | --- |
| G-CSF | IL-17A |
| GM-CSF | IL-23p19 |
| GRO-α/CXCL1/KC | IL-27 |
| HGF | IP-10/CXCL10/CRG-2 |
| IFN-γ | MCP-1/CCL2/JE |
| IL-1 α | M-CSF |
| IL-1 β | MMP-3 |
| IL-3 | MMP-9 |
| IL-6 | RAGE |
| IL-10 | RANTES/CCL5 |
| IL-12 (p70) | S100A8 |
| IL-13 | VEGF |
| IL-16 | Thrombospondin-4 |
| TNF- α |  |

**Table S5. Cytokines selected for Bioplex analysis**

**
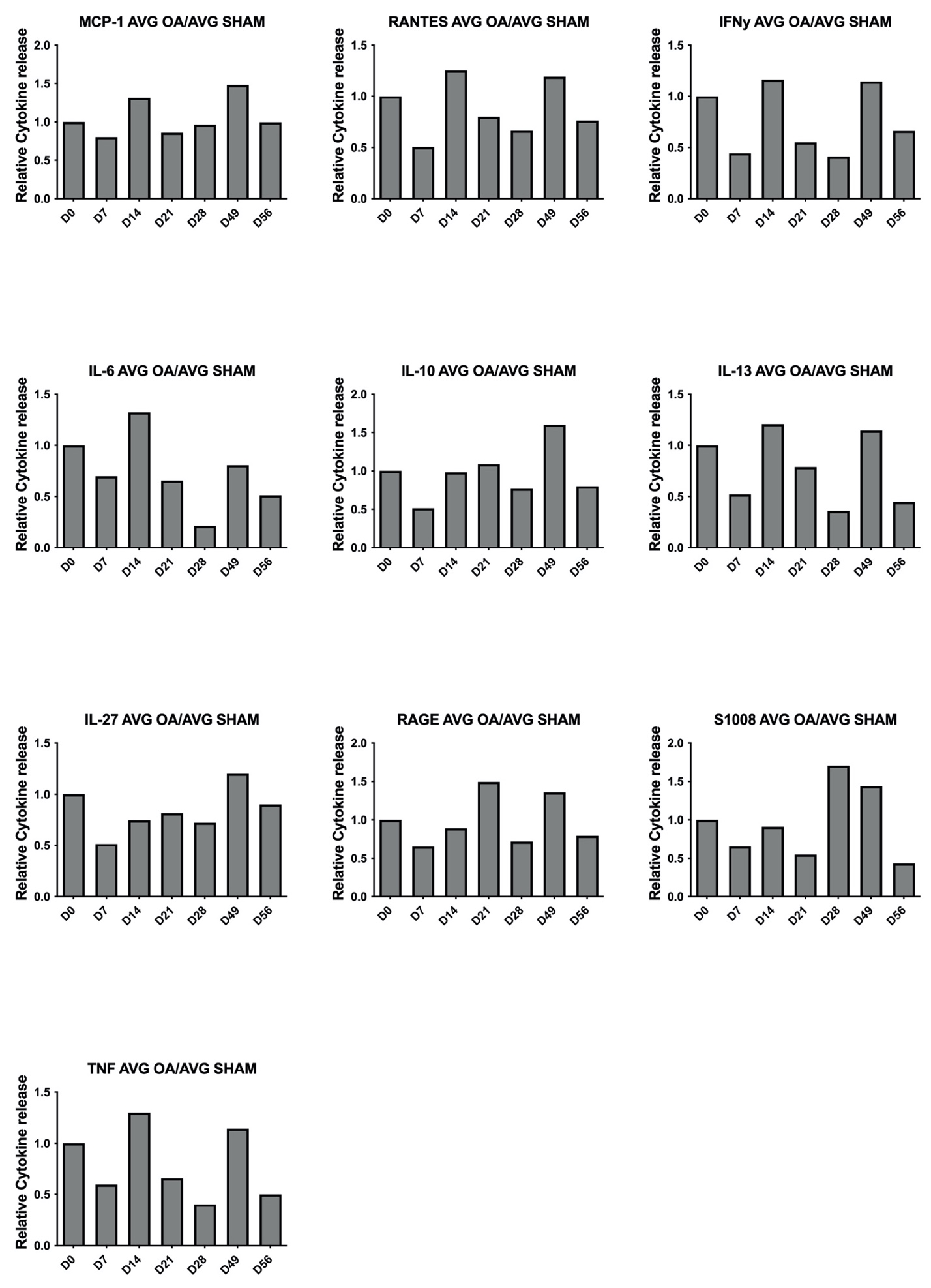
**

**Fig. S5. Analysis of cytokine levels of a panel of OA associated cytokines over 56 days following CIOA in Sham versus CIOA.** The concentration of each cytokine is shown relative to the CIOA group, with the average concentration in the CIOA group divided by the average concentration of the Sham group.


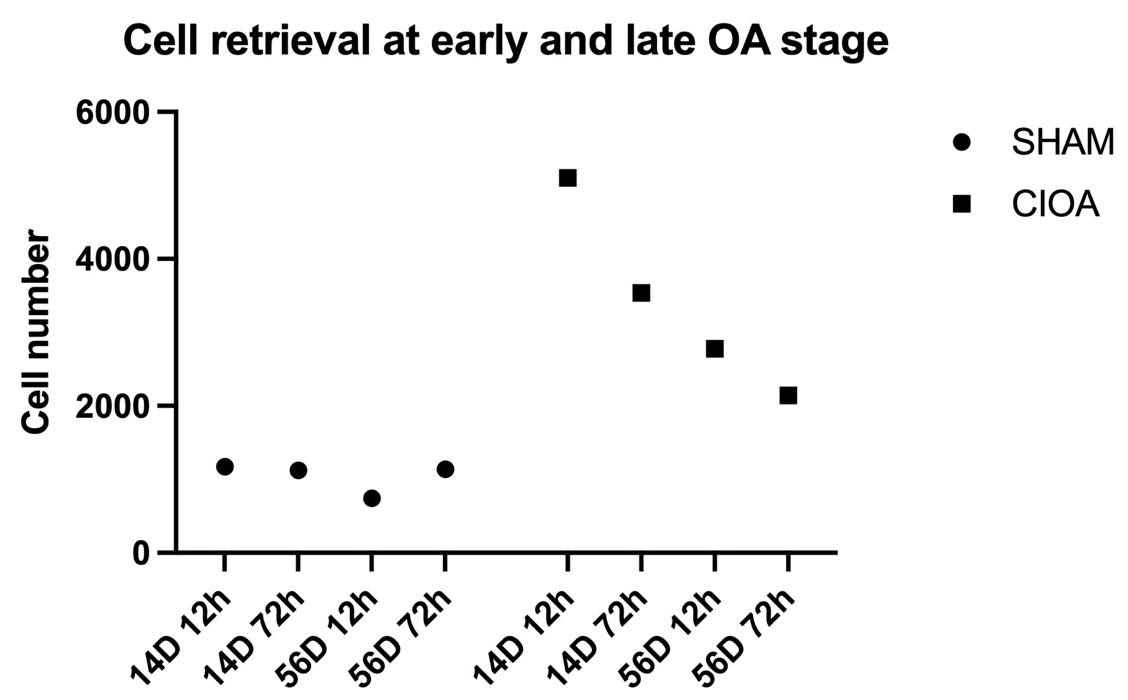


**Fig. S6. Number of GFP+MSCs retrieved from CIOA and SHAM joints for RNA sequencing.** For each time point cells were pooled and retrieved from n=8 mice (16 knee joints).


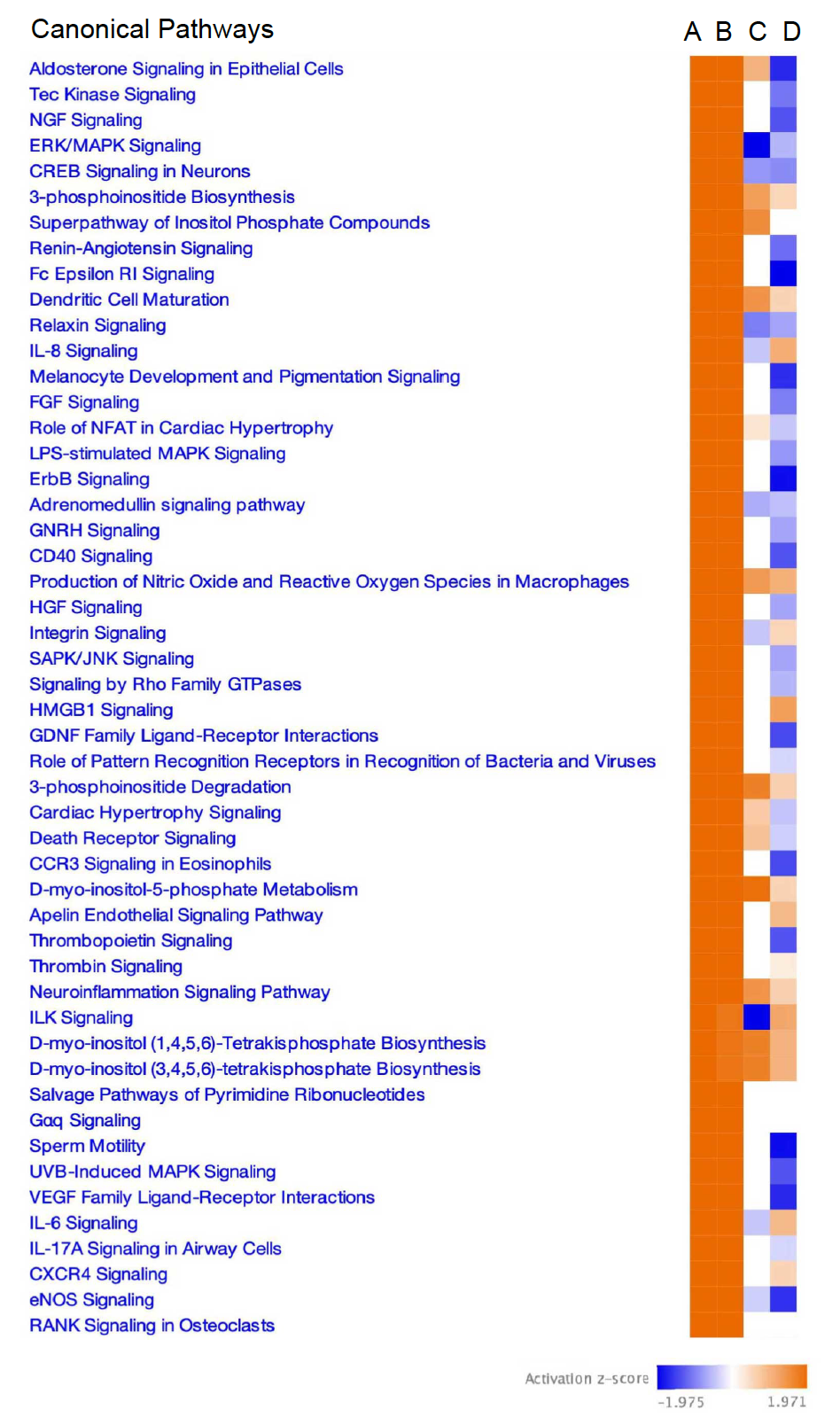


**Fig. S7. Heatmap of activated canonical pathways.** Retrieved MSCs at early 12-hour (A, B) and late 3-day time-points (C, D), showing activation of the majority of pathways at the early retrieval time point of 12 hours.

**
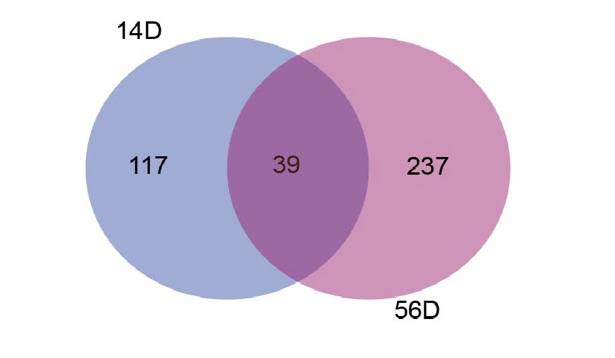
**

**Fig. S8 Overlay of Predicted secreted elements in retrieved MSCs at D14 and D56** Venn diagram representing time-point specific and common proteins secreted by retrieved MSCs, indicating a higher number of elements at 56D.


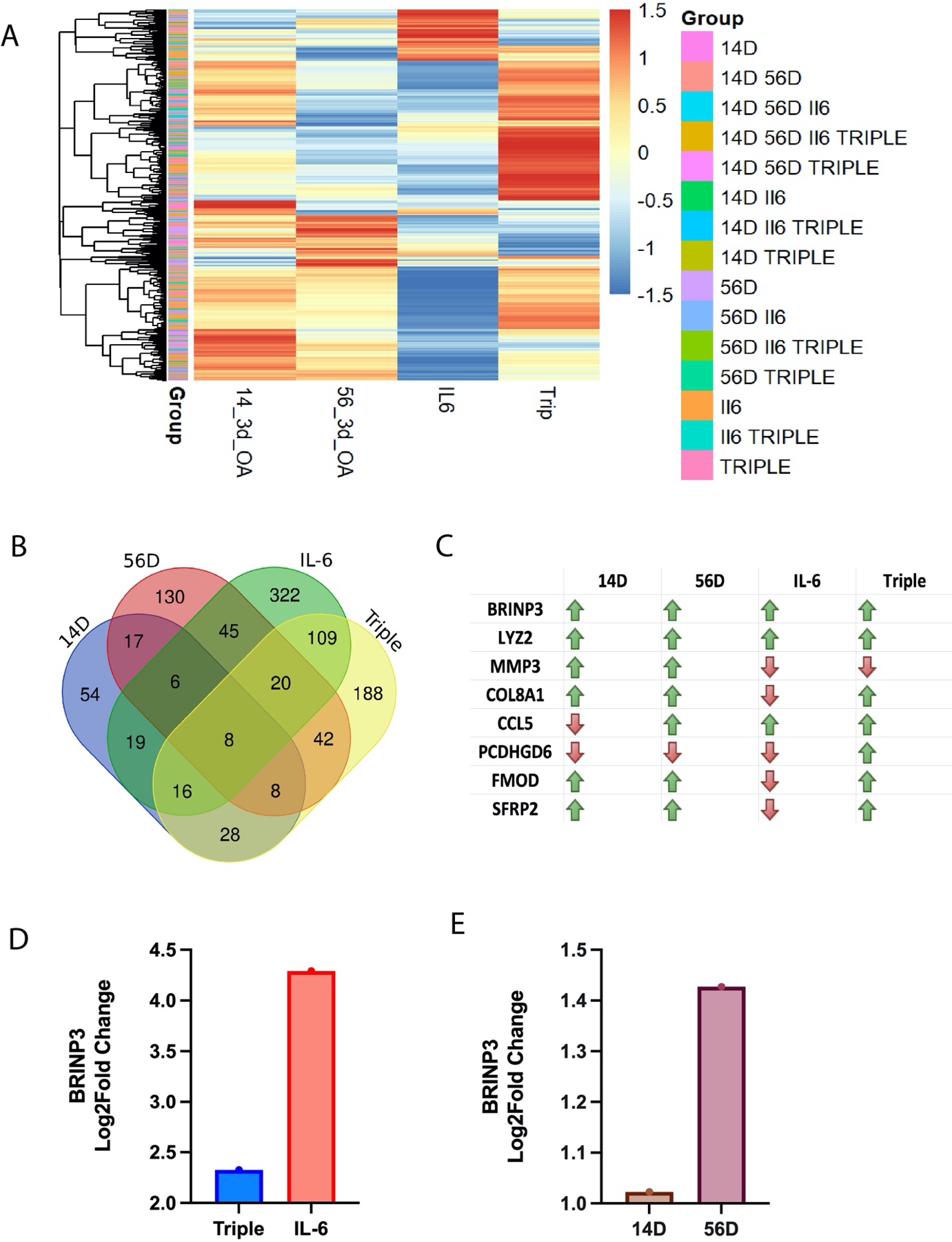


**Fig. S9.** **Common expressed genes in licensed and retrieved MSCs.** (**A**) Heatmap representing a hierarchal comparison of the predicted secreted elements from three-day retrieved cells at 14D and 56D CIOA and in vitro licensed cells with IL-6 and the triple combination (IL-6, MCP-1 and IFN-γ) licensing. (**B**) Venn diagram comparison of the predicted secretome of retrieved cells and licensed MSCs indicate eight common elements (**C**) Up-regulation and down-regulation of common genes expressed in the predicted secretome of retrieved and licensed MSCs (**D**) RNA Sequencing expression of BRINP3 in (**D**) *in vitro* licensed GFP+MSCs with IL-6 and triple combination vs naïve GFP+MSCs and in (**E)** three-day retrieved GFP+MSCs at 14D and 56D CIOA vs SHAM

| #GeneSymbol | GeneName | Log2FC |
| --- | --- | --- |
| Prokr1 | prokineticin receptor 1 | 4,46194 |
| F13a1 | coagulation factor XIII, A1 subunit | 4,07296 |
| Grem1 | gremlin 1, DAN family BMP antagonist | 3,64384 |
| Megf6 | multiple EGF-like-domains 6 | 3,43041 |
| Atp6v0d2 | ATPase, H+ transporting, lysosomal V0 subunit D2 | 3,41636 |
| Ngp | neutrophilic granule protein | 3,35971 |
| Fam198a | family with sequence similarity 198, member A | 3,27547 |
| Pcdhga1 | protocadherin gamma subfamily A, 1 | 3,13658 |
| Ccser1 | coiled-coil serine rich 1 | 3,11062 |
| Xpnpep2 | X-prolyl aminopeptidase (aminopeptidase P) 2, membrane-bound | 3,02054 |
| Trp53inp1 | transformation related protein 53 inducible nuclear protein 1 | 2,8636 |
| Oas2 | 2'-5' oligoadenylate synthetase 2 | 2,83016 |
| Rnd1 | Rho family GTPase 1 | 2,7993 |
| Fgl2 | fibrinogen-like protein 2 | 2,77837 |
| F8 | coagulation factor VIII | 2,72218 |
| Gm14308 | predicted gene 14308 | 2,68641 |
| Mmp12 | matrix metallopeptidase 12 | 2,62773 |
| Mmp13 | matrix metallopeptidase 13 | 2,61103 |
| Prg4 | proteoglycan 4 (megakaryocyte stimulating factor, articular superficial zone protein) | 2,60831 |
| Gdpd2 | glycerophosphodiester phosphodiesterase domain containing 2 | 2,59065 |
| Neurl1b | neuralized E3 ubiquitin protein ligase 1B | 2,56539 |
| Cmpk2 | cytidine monophosphate (UMP-CMP) kinase 2, mitochondrial | 2,56079 |
| Ccl20 | chemokine (C-C motif) ligand 20 | 2,55258 |
| Angptl1 | angiopoietin-like 1 | 2,54484 |
| Dnajc27 | DnaJ heat shock protein family (Hsp40) member C27 | 2,52886 |
| 1500015O10Rik | RIKEN cDNA 1500015O10 gene | 2,52443 |
| Acp6 | acid phosphatase 6, lysophosphatidic | 2,50574 |
| Zfp420 | zinc finger protein 420 | 2,48501 |
| Egfl6 | EGF-like-domain, multiple 6 | 2,478 |
| Bnip3l-ps | BCL2/adenovirus E1B interacting protein 3-like, pseudogene | 2,45969 |
| Cdkn1c | cyclin-dependent kinase inhibitor 1C (P57) | 2,45235 |
| Nos2 | nitric oxide synthase 2, inducible | 2,4331 |
| Ifit3 | interferon-induced protein with tetratricopeptide repeats 3 | 2,42527 |
| Gpnmb | glycoprotein (transmembrane) nmb | 2,42514 |
| Fbln7 | fibulin 7 | 2,40116 |
| Apod | apolipoprotein D | 2,37814 |
| Chodl | chondrolectin | 2,36039 |
| Elmo1 | engulfment and cell motility 1 | 2,35538 |
| Zfp59 | zinc finger protein 59 | 2,34563 |
| Fam71f2 | family with sequence similarity 71, member F2 | 2,33429 |
| Fndc1 | fibronectin type III domain containing 1 | 2,33131 |
| Camsap3 | calmodulin regulated spectrin-associated protein family, member 3 | 2,33072 |
| Ifi207 | interferon activated gene 207 | 2,31823 |
| Slc16a2 | solute carrier family 16 (monocarboxylic acid transporters), member 2 | 2,3028 |
| Akr1c18 | aldo-keto reductase family 1, member C18 | 2,30042 |
| Oas1b | 2'-5' oligoadenylate synthetase 1B | 2,2966 |
| Zfp781 | zinc finger protein 781 | 2,28503 |
| Gbp5 | guanylate binding protein 5 | 2,27757 |
| Mmp2 | matrix metallopeptidase 2 | 2,21567 |

| Gm4951 | predicted gene 4951 | 2,21413 |
| --- | --- | --- |
| Naalad2 | N-acetylated alpha-linked acidic dipeptidase 2 | 2,20552 |
| Mmp3 | matrix metallopeptidase 3 | 2,19535 |
| Rassf2 | Ras association (RalGDS/AF-6) domain family member 2 | 2,19313 |
| Ggt5 | gamma-glutamyltransferase 5 | 2,18908 |
| Igfbp5 | insulin-like growth factor binding protein 5 | 2,18433 |
| Ddit4 | DNA-damage-inducible transcript 4 | 2,17282 |
| Pcdhb9 | protocadherin beta 9 | 2,17245 |
| Ppargc1a | peroxisome proliferative activated receptor, gamma, coactivator 1 alpha | 2,15562 |
| Igf1 | insulin-like growth factor 1 | 2,12985 |
| Cdc42bpg | CDC42 binding protein kinase gamma (DMPK-like) | 2,12138 |
| Htra4 | HtrA serine peptidase 4 | 2,12138 |
| Ptx4 | pentraxin 4 | 2,12138 |
| Ctla2a | cytotoxic T lymphocyte-associated protein 2 alpha | 2,11018 |
| Il6 | interleukin 6 | 2,06588 |
| Zfp27 | zinc finger protein 27 | 2,05549 |
| Il27ra | interleukin 27 receptor, alpha | 2,05035 |
| Usp18 | ubiquitin specific peptidase 18 | 2,0461 |
| Zfp239 | zinc finger protein 239 | 2,01545 |
| Pla2g4b | phospholipase A2, group IVB (cytosolic) | 2,01347 |
| Fam102a | family with sequence similarity 102, member A | 2,01199 |
| Pcdhb14 | protocadherin beta 14 | 2,00804 |
| Zfp185 | zinc finger protein 185 | 1,99468 |
| Vtn | vitronectin | 1,97564 |
| F830016B08Rik | RIKEN cDNA F830016B08 gene | 1,96627 |
| Myo7a | myosin VIIA | 1,96214 |
| Iigp1 | interferon inducible GTPase 1 | 1,95898 |
| Klhl10 | kelch-like 10 | 1,95635 |
| Map3k8 | mitogen-activated protein kinase kinase kinase 8 | 1,95389 |
| Mfsd6 | major facilitator superfamily domain containing 6 | 1,93677 |
| Bmper | BMP-binding endothelial regulator | 1,92203 |
| Ctsw | cathepsin W | 1,91696 |
| Sfrp2 | secreted frizzled-related protein 2 | 1,91013 |
| Mx1 | MX dynamin-like GTPase 1 | 1,87973 |
| Usp27x | ubiquitin specific peptidase 27, X chromosome | 1,87646 |
| Nr1h4 | nuclear receptor subfamily 1, group H, member 4 | 1,87646 |
| Il33 | interleukin 33 | 1,87473 |
| Rad51d | RAD51 paralog D | 1,87002 |
| Col8a2 | collagen, type VIII, alpha 2 | 1,8636 |
| Sfrp4 | secreted frizzled-related protein 4 | 1,86068 |
| Plcl2 | phospholipase C-like 2 | 1,85907 |
| Rcan2 | regulator of calcineurin 2 | 1,85064 |
| Zfp867 | zinc finger protein 867 | 1,85024 |
| Hs6st2 | heparan sulfate 6-O-sulfotransferase 2 | 1,84515 |
| Aoc3 | amine oxidase, copper containing 3 | 1,84514 |
| Hilpda | hypoxia inducible lipid droplet associated | 1,8411 |
| Itgb2 | integrin beta 2 | 1,8348 |
| Fam131b | family with sequence similarity 131, member B | 1,83402 |
| Hk2 | hexokinase 2 | 1,82919 |
| Tnfsf9 | tumor necrosis factor (ligand) superfamily, member 9 | 1,81989 |
| Fyb2 | FYN binding protein 2 | 1,81352 |

**Table S6. Top 100 up-regulated DEGs in retrieved MSCs (14D CIOA vs 14D SHAM).**

| #GeneSymbol | GeneName | Log2FC |
| --- | --- | --- |
| Uba52 | ubiquitin A-52 residue ribosomal protein fusion product 1 | -7,32197 |
| Acan | aggrecan | -3,69007 |
| Olr1 | oxidized low density lipoprotein (lectin-like) receptor 1 | -3,60373 |
| Dpm3 | dolichyl-phosphate mannosyltransferase polypeptide 3 | -2,9975 |
| Ndufb7 | NADH:ubiquinone oxidoreductase subunit B7 | -2,88509 |
| Gtpbp6 | GTP binding protein 6 (putative) | -2,78429 |
| Wdr62 | WD repeat domain 62 | -2,74532 |
| Fam19a5 | family with sequence similarity 19, member A5 | -2,74356 |
| Elob | elongin B | -2,7287 |
| Lrrc29 | leucine rich repeat containing 29 | -2,72311 |
| Ccdc124 | coiled-coil domain containing 124 | -2,70284 |
| Dohh | deoxyhypusine hydroxylase/monooxygenase | -2,64849 |
| Timm13 | translocase of inner mitochondrial membrane 13 | -2,63643 |
| Ltbp4 | latent transforming growth factor beta binding protein 4 | -2,63024 |
| Bola2 | bolA-like 2 (E. coli) | -2,60279 |
| Pbp2 | phosphatidylethanolamine binding protein 2 | -2,59269 |
| Pcdhgb6 | protocadherin gamma subfamily B, 6 | -2,57926 |
| Znhit2 | zinc finger, HIT domain containing 2 | -2,56475 |
| P2ry2 | purinergic receptor P2Y, G-protein coupled 2 | -2,54545 |
| Lgr6 | leucine-rich repeat-containing G protein-coupled receptor 6 | -2,50834 |
| Angptl7 | angiopoietin-like 7 | -2,48176 |
| Edf1 | endothelial differentiation-related factor 1 | -2,47101 |
| Olfm2 | olfactomedin 2 | -2,43324 |
| Lrfn4 | leucine rich repeat and fibronectin type III domain containing 4 | -2,4302 |
| Mt2 | metallothionein 2 | -2,41012 |
| Hspb1 | heat shock protein 1 | -2,40978 |
| Unc13c | unc-13 homolog C | -2,4045 |
| Chpf | chondroitin polymerizing factor | -2,39721 |
| Slurp1 | secreted Ly6/Plaur domain containing 1 | -2,38753 |
| Zfp763 | zinc finger protein 763 | -2,38073 |
| Junb | jun B proto-oncogene | -2,37058 |
| Tppp3 | tubulin polymerization-promoting protein family member 3 | -2,31318 |
| C1qtnf2 | C1q and tumor necrosis factor related protein 2 | -2,31208 |
| Chtf18 | CTF18, chromosome transmission fidelity factor 18 | -2,30891 |
| L1cam | L1 cell adhesion molecule | -2,29031 |
| Crip2 | cysteine rich protein 2 | -2,27929 |
| Pold1 | polymerase (DNA directed), delta 1, catalytic subunit | -2,25264 |
| Fam213a | family with sequence similarity 213, member A | -2,25135 |
| Acsbg1 | acyl-CoA synthetase bubblegum family member 1 | -2,24772 |
| Cep131 | centrosomal protein 131 | -2,23732 |
| Kcnk5 | potassium channel, subfamily K, member 5 | -2,21841 |
| Tpgs1 | tubulin polyglutamylase complex subunit 1 | -2,21367 |
| Zfp82 | zinc finger protein 82 | -2,20889 |
| Smad9 | SMAD family member 9 | -2,20806 |
| Ccnd1 | cyclin D1 | -2,18187 |
| Mfsd3 | major facilitator superfamily domain containing 3 | -2,16819 |
| Aldh1a3 | aldehyde dehydrogenase family 1, subfamily A3 | -2,15602 |
| Lamb2 | laminin, beta 2 | -2,14246 |
| Acad10 | acyl-Coenzyme A dehydrogenase family, member 10 | -2,12345 |
| Tmem26 | transmembrane protein 26 | -2,12345 |
| Fam19a2 | family with sequence similarity 19, member A2 | -2,11672 |
| Trib3 | tribbles pseudokinase 3 | -2,11016 |
| Fgfr3 | fibroblast growth factor receptor 3 | -2,09753 |
| Acpp | acid phosphatase, prostate | -2,09207 |
| Fancd2 | Fanconi anemia, complementation group D2 | -2,09152 |
| Icam5 | intercellular adhesion molecule 5, telencephalin | -2,08095 |
| Hspa1a | heat shock protein 1A | -2,0742 |
| Thap7 | THAP domain containing 7 | -2,06643 |
| Slc12a9 | solute carrier family 12 (potassium/chloride transporters), member 9 | -2,06569 |
| Stra6 | stimulated by retinoic acid gene 6 | -2,06248 |
| Rnaseh2c | ribonuclease H2, subunit C | -2,0614 |
| Cspg4 | chondroitin sulfate proteoglycan 4 | -2,05874 |
| Gchfr | GTP cyclohydrolase I feedback regulator | -2,05575 |
| Psmb10 | proteasome (prosome, macropain) subunit, beta type 10 | -2,0538 |
| Igfbp6 | insulin-like growth factor binding protein 6 | -2,04166 |
| Ccdc141 | coiled-coil domain containing 141 | -2,01787 |
| Card10 | caspase recruitment domain family, member 10 | -2,0167 |
| Erdr1 | erythroid differentiation regulator 1 | -2,00335 |
| Zfp189 | zinc finger protein 189 | -2,00018 |
| Hspbp1 | HSPA (heat shock 70kDa) binding protein, cytoplasmic cochaperone 1 | -2,00016 |
| Lgals7 | lectin, galactose binding, soluble 7 | -1,99739 |
| Klrg2 | killer cell lectin-like receptor subfamily G, member 2 | -1,98652 |
| Polr2f | polymerase (RNA) II (DNA directed) polypeptide F | -1,95696 |
| Zfp414 | zinc finger protein 414 | -1,92811 |
| Jmjd4 | jumonji domain containing 4 | -1,92514 |
| Pcolce2 | procollagen C-endopeptidase enhancer 2 | -1,91977 |
| Cenpm | centromere protein M | -1,9166 |
| Ptpn22 | protein tyrosine phosphatase, non-receptor type 22 (lymphoid) | -1,89205 |
| Glis1 | GLIS family zinc finger 1 | -1,88842 |
| Zswim3 | zinc finger SWIM-type containing 3 | -1,88834 |
| Slc6a17 | solute carrier family 6 (neurotransmitter transporter), member 17 | -1,88775 |
| Lmna | lamin A | -1,88056 |
| Csnk1g2 | casein kinase 1, gamma 2 | -1,8566 |
| Odaph | odontogenesis associated phosphoprotein | -1,85124 |
| Ubtd1 | ubiquitin domain containing 1 | -1,84807 |
| Kcnn4 | potassium intermediate/small conductance calcium-activated channel, subfamily N, member 4 | -1,84666 |
| Fzr1 | fizzy and cell division cycle 20 related 1 | -1,84279 |
| Scrn2 | secernin 2 | -1,83715 |
| Tspo | translocator protein | -1,82875 |
| Ndufs7 | NADH:ubiquinone oxidoreductase core subunit S7 | -1,82691 |
| Aldh16a1 | aldehyde dehydrogenase 16 family, member A1 | -1,81978 |
| Mif | macrophage migration inhibitory factor (glycosylation-inhibiting factor) | -1,81817 |
| Ndufa11 | NADH:ubiquinone oxidoreductase subunit A11 | -1,81742 |
| Emilin1 | elastin microfibril interfacer 1 | -1,81466 |
| Ppbp | pro-platelet basic protein | -1,81348 |
| Plekhh3 | pleckstrin homology domain containing, family H (with MyTH4 domain) member 3 | -1,80605 |
| Orai1 | ORAI calcium release-activated calcium modulator 1 | -1,80563 |
| Trp53i13 | transformation related protein 53 inducible protein 13 | -1,79746 |
| Esco2 | establishment of sister chromatid cohesion N-acetyltransferase 2 | -1,79543 |
| Plppr4 | phospholipid phosphatase related 4 | -1,79478 |

**Table S7. Top 100 down-regulated DEGs in retrieved MSCs (14D CIOA vs 14D SHAM)**

| #GeneSymbol | GeneName | Log2FC |
| --- | --- | --- |
| Ctss | cathepsin S | 6,14732 |
| Lcn2 | lipocalin 2 | 6,05642 |
| C1qb | complement component 1, q subcomponent, beta polypeptide | 6,02481 |
| Pgf | placental growth factor | 5,1662 |
| C1qc | complement component 1, q subcomponent, C chain | 5,14797 |
| Arg1 | arginase, liver | 5,10158 |
| Cxcl3 | chemokine (C-X-C motif) ligand 3 | 5,02552 |
| Scn7a | sodium channel, voltage-gated, type VII, alpha | 4,99685 |
| Gm4724 | predicted gene 4724 | 4,96816 |
| Serpina3n | serine (or cysteine) peptidase inhibitor, clade A, member 3N | 4,95951 |
| Susd2 | sushi domain containing 2 | 4,87606 |
| Cpne4 | copine IV | 4,78674 |
| Odaph | odontogenesis associated phosphoprotein | 4,77609 |
| Pcdhgb2 | protocadherin gamma subfamily B, 2 | 4,71834 |
| Zfp420 | zinc finger protein 420 | 4,71811 |
| Penk | preproenkephalin | 4,67959 |
| Apcdd1 | adenomatosis polyposis coli down-regulated 1 | 4,6746 |
| Tyrobp | TYRO protein tyrosine kinase binding protein | 4,60864 |
| Zfp955b | zinc finger protein 955B | 4,60864 |
| Moxd1 | monooxygenase, DBH-like 1 | 4,57232 |
| Snx11 | sorting nexin 11 | 4,5135 |
| Mgat4a | mannoside acetylglucosaminyltransferase 4, isoenzyme A | 4,50043 |
| Alox5ap | arachidonate 5-lipoxygenase activating protein | 4,47985 |
| Gbp9 | guanylate-binding protein 9 | 4,4382 |
| Ms4a6d | membrane-spanning 4-domains, subfamily A, member 6D | 4,4378 |
| Hexim2 | hexamethylene bis-acetamide inducible 2 | 4,4271 |
| Csf3 | colony stimulating factor 3 (granulocyte) | 4,41153 |
| Gtpbp8 | GTP-binding protein 8 (putative) | 4,37722 |
| Cd177 | CD177 antigen | 4,36111 |
| Serping1 | serine (or cysteine) peptidase inhibitor, clade G, member 1 | 4,36012 |
| Gm28042 | predicted gene, 28042 | 4,29198 |
| Tarsl2 | threonyl-tRNA synthetase-like 2 | 4,24399 |
| Fcnb | ficolin B | 4,2069 |
| Lyz2 | lysozyme 2 | 4,18611 |
| Gm12258 | predicted gene 12258 | 4,18164 |
| B3galt4 | UDP-Gal:betaGlcNAc beta 1,3-galactosyltransferase, polypeptide 4 | 4,12975 |
| C1qa | complement component 1, q subcomponent, alpha polypeptide | 4,11649 |
| Cd300c2 | CD300C molecule 2 | 4,11649 |
| Zfp748 | zinc finger protein 748 | 4,11363 |
| Laptm5 | lysosomal-associated protein transmembrane 5 | 4,10309 |
| Enox1 | ecto-NOX disulfide-thiol exchanger 1 | 4,08959 |
| D3Ertd751e | DNA segment, Chr 3, ERATO Doi 751, expressed | 4,08959 |
| Eif5a2 | eukaryotic translation initiation factor 5A2 | 4,02287 |
| Cxcr6 | chemokine (C-X-C motif) receptor 6 | 4,02003 |
| Zfp790 | zinc finger protein 790 | 3,98951 |
| C5ar1 | complement component 5a receptor 1 | 3,97664 |
| A430033K04Rik | RIKEN cDNA A430033K04 gene | 3,97657 |
| Cxcl9 | chemokine (C-X-C motif) ligand 9 | 3,97136 |
| Sfrp1 | secreted frizzled-related protein 1 | 3,96821 |
| Rnf113a2 | ring finger protein 113A2 | 3,96188 |
| Tctn3 | tectonic family member 3 | 3,93965 |
| Slc25a26 | solute carrier family 25 (mitochondrial carrier, phosphate carrier), member 26 | 3,92865 |
| Kif26b | kinesin family member 26B | 3,91005 |
| Bbs12 | Bardet-Biedl syndrome 12 (human) | 3,90273 |
| Thnsl2 | threonine synthase-like 2 (bacterial) | 3,85067 |
| Fkrp | fukutin related protein | 3,85036 |
| Prdm9 | PR domain containing 9 | 3,8462 |
| Thsd1 | thrombospondin, type I, domain 1 | 3,83803 |
| Cdh23 | cadherin 23 (otocadherin) | 3,82177 |
| Spry3 | sprouty RTK signaling antagonist 3 | 3,82177 |
| Zfp781 | zinc finger protein 781 | 3,82177 |
| 1700066M21Rik | RIKEN cDNA 1700066M21 gene | 3,81831 |
| Fmod | fibromodulin | 3,79743 |
| Coro1a | coronin, actin binding protein 1A | 3,7887 |
| Steap4 | STEAP family member 4 | 3,77971 |
| Cdhr1 | cadherin-related family member 1 | 3,77188 |
| Lilrb4a | leukocyte immunoglobulin-like receptor, subfamily B, member 4A | 3,77188 |
| Ccdc39 | coiled-coil domain containing 39 | 3,73763 |
| Pf4 | platelet factor 4 | 3,73763 |
| Apln | apelin | 3,71607 |
| Chrd | chordin | 3,70245 |
| Cyb561d1 | cytochrome b-561 domain containing 1 | 3,68485 |
| Sema4f | sema domain, immunoglobulin domain (Ig), TM domain, and short cytoplasmic domain | 3,68469 |
| Hck | hemopoietic cell kinase | 3,68469 |
| Havcr2 | hepatitis A virus cellular receptor 2 | 3,64828 |
| Tcfl5 | transcription factor-like 5 (basic helix-loop-helix) | 3,64828 |
| Iba57 | IBA57 homolog, iron-sulfur cluster assembly | 3,64828 |
| Pcdhb16 | protocadherin beta 16 | 3,64517 |
| Duoxa1 | dual oxidase maturation factor 1 | 3,63518 |
| Pcsk9 | proprotein convertase subtilisin/kexin type 9 | 3,62973 |
| Trmt12 | tRNA methyltranferase 12 | 3,62547 |
| Zfp280b | zinc finger protein 280B | 3,61137 |
| Ddias | DNA damage-induced apoptosis suppressor | 3,61094 |
| Mylpf | myosin light chain, phosphorylatable, fast skeletal muscle | 3,59189 |
| Thbs4 | thrombospondin 4 | 3,58663 |
| Pcsk6 | proprotein convertase subtilisin/kexin type 6 | 3,5726 |
| Arhgef6 | Rac/Cdc42 guanine nucleotide exchange factor (GEF) 6 | 3,56318 |
| Mamdc2 | MAM domain containing 2 | 3,55957 |
| Wnt7b | wingless-type MMTV integration site family, member 7B | 3,55718 |
| B3gat2 | beta-1,3-glucuronyltransferase 2 (glucuronosyltransferase S) | 3,55302 |
| Il1b | interleukin 1 beta | 3,53321 |
| Ubd | ubiquitin D | 3,53321 |
| Chrdl1 | chordin-like 1 | 3,52242 |
| Zfp995 | zinc finger protein 995 | 3,51423 |
| Cd48 | CD48 antigen | 3,51309 |
| Plac1 | placental specific protein 1 | 3,51309 |
| Comp | cartilage oligomeric matrix protein | 3,50445 |
| Fabp4 | fatty acid binding protein 4, adipocyte | 3,49271 |
| Cotl1 | coactosin-like 1 (Dictyostelium) | 3,48679 |
| Cd83 | CD83 antigen | 3,47204 |

**Table S8.** **Top 100 up-regulated DEGs in retrieved MSCs (56D CIOA vs 56D SHAM*)***

| #GeneSymbol | GeneName | Log2FC |
| --- | --- | --- |
| 1700030J22Rik | RIKEN cDNA 1700030J22 gene | -5,10552 |
| Acot11 | acyl-CoA thioesterase 11 | -5,05112 |
| Zbtb14 | zinc finger and BTB domain containing 14 | -4,67226 |
| Zbtb3 | zinc finger and BTB domain containing 3 | -4,62415 |
| Snx32 | sorting nexin 32 | -4,56498 |
| Efcc1 | EF hand and coiled-coil domain containing 1 | -4,51226 |
| Ube2cbp | ubiquitin-conjugating enzyme E2C binding protein | -4,46883 |
| Pcdhb4 | protocadherin beta 4 | -4,37138 |
| Kctd19 | potassium channel tetramerisation domain containing 19 | -4,06474 |
| Tspan32 | tetraspanin 32 | -4,01509 |
| Tppp | tubulin polymerization promoting protein | -3,96367 |
| Cacng1 | calcium channel, voltage-dependent, gamma subunit 1 | -3,85497 |
| Exd2 | exonuclease 3'-5' domain containing 2 | -3,8233 |
| Iyd | iodotyrosine deiodinase | -3,78264 |
| Cd72 | CD72 antigen | -3,76773 |
| Igflr1 | IGF-like family receptor 1 | -3,76773 |
| Samd12 | sterile alpha motif domain containing 12 | -3,76773 |
| Depdc7 | DEP domain containing 7 | -3,75266 |
| Ppl | periplakin | -3,75041 |
| Avil | advillin | -3,74685 |
| Dnajc22 | DnaJ heat shock protein family (Hsp40) member C22 | -3,72204 |
| Zfp493 | zinc finger protein 493 | -3,70445 |
| Ahrr | aryl-hydrocarbon receptor repressor | -3,69726 |
| Napsa | napsin A aspartic peptidase | -3,67485 |
| Cct6b | chaperonin containing Tcp1, subunit 6b (zeta) | -3,67485 |
| Dynap | dynactin associated protein | -3,64253 |
| Hspa1l | heat shock protein 1-like | -3,62609 |
| Lrrc71 | leucine rich repeat containing 71 | -3,59263 |
| Apba1 | amyloid beta (A4) precursor protein binding, family A, member 1 | -3,58183 |
| Cntnap4 | contactin associated protein-like 4 | -3,55746 |
| Pth1r | parathyroid hormone 1 receptor | -3,52331 |
| Lrba | LPS-responsive beige-like anchor | -3,51222 |
| Ism1 | isthmin 1, angiogenesis inhibitor | -3,50545 |
| Bcan | brevican | -3,48735 |
| Kcnt2 | potassium channel, subfamily T, member 2 | -3,48201 |
| U90926 | cDNA sequence U90926 | -3,46904 |
| Trim43a | tripartite motif-containing 43A | -3,46904 |
| 1700012B09Rik | RIKEN cDNA 1700012B09 gene | -3,45048 |
| Tmc4 | transmembrane channel-like gene family 4 | -3,43226 |
| Prnd | prion like protein doppel | -3,43169 |
| Fam163a | family with sequence similarity 163, member A | -3,41265 |
| Rragd | Ras-related GTP binding D | -3,39627 |
| Olfr99 | olfactory receptor 99 | -3,39336 |
| Fbxl2 | F-box and leucine-rich repeat protein 2 | -3,38342 |
| Cxcl13 | chemokine (C-X-C motif) ligand 13 | -3,37378 |
| Nek11 | NIMA (never in mitosis gene a)-related expressed kinase 11 | -3,37378 |
| Fgf2 | fibroblast growth factor 2 | -3,35497 |
| Zfp296 | zinc finger protein 296 | -3,35397 |
| Ptgdr2 | prostaglandin D2 receptor 2 | -3,35397 |
| AI481877 | expressed sequence AI481877 | -3,35397 |
| Coch | cochlin | -3,31346 |
| Cela3b | chymotrypsin-like elastase family, member 3B | -3,31346 |
| Slc25a29 | solute carrier family 25 (mitochondrial carrier, palmitoylcarnitine transporter), member 29 | -3,2928 |
| Dpysl5 | dihydropyrimidinase-like 5 | -3,28016 |
| Acad11 | acyl-Coenzyme A dehydrogenase family, member 11 | -3,26172 |
| Ptx4 | pentraxin 4 | -3,24616 |
| Bglap3 | bone gamma-carboxyglutamate protein 3 | -3,22892 |
| Dnase2b | deoxyribonuclease II beta | -3,18471 |
| Nfe2l3 | nuclear factor, erythroid derived 2, like 3 | -3,18471 |
| Ifna4 | interferon alpha 4 | -3,18471 |
| Eri2 | exoribonuclease 2 | -3,17447 |
| Ttll11 | tubulin tyrosine ligase-like family, member 11 | -3,16208 |
| Iqck | IQ motif containing K | -3,1527 |
| Zfp941 | zinc finger protein 941 | -3,15052 |
| Pla2g4b | phospholipase A2, group IVB (cytosolic) | -3,1406 |
| Prkcb | protein kinase C, beta | -3,13594 |
| Vmn2r1 | vomeronasal 2, receptor 1 | -3,11575 |
| Sv2a | synaptic vesicle glycoprotein 2 a | -3,11495 |
| Mmab | methylmalonic aciduria (cobalamin deficiency) cblB type homolog (human) | -3,10925 |
| Lipc | lipase, hepatic | -3,06789 |
| Sec1 | secretory blood group 1 | -3,06789 |
| Coro2a | coronin, actin binding protein 2A | -3,04335 |
| Rbm12b1 | RNA binding motif protein 12 B1 | -3,04335 |
| Capn12 | calpain 12 | -3,04335 |
| Slc14a2 | solute carrier family 14 (urea transporter), member 2 | -3,01837 |
| Tle4 | transducin-like enhancer of split 4 | -3,01609 |
| Zfp599 | zinc finger protein 599 | -3,01096 |
| Omd | osteomodulin | -2,99825 |
| Mob3b | MOB kinase activator 3B | -2,96687 |
| Xkr8 | X-linked Kx blood group related 8 | -2,94963 |
| Fbxo44 | F-box protein 44 | -2,945 |
| Lrrc46 | leucine rich repeat containing 46 | -2,94078 |
| Slc2a13 | solute carrier family 2 (facilitated glucose transporter), member 13 | -2,92384 |
| Evi2b | ecotropic viral integration site 2b | -2,91396 |
| Kcna4 | potassium voltage-gated channel, shaker-related subfamily, member 4 | -2,90568 |
| Xrcc2 | X-ray repair complementing defective repair in Chinese hamster cells 2 | -2,89839 |
| Spats1 | spermatogenesis associated, serine-rich 1 | -2,88664 |
| Adtrp | androgen dependent TFPI regulating protein | -2,88664 |
| Tnfsf13 | tumor necrosis factor (ligand) superfamily, member 13 | -2,88664 |
| Kdm8 | lysine (K)-specific demethylase 8 | -2,86564 |
| Vwc2l | von Willebrand factor C domain-containing protein 2-like | -2,85878 |
| Glt28d2 | glycosyltransferase 28 domain containing 2 | -2,84294 |
| Il1rap | interleukin 1 receptor accessory protein | -2,83394 |
| Ssc4d | scavenger receptor cysteine rich family, 4 domains | -2,83037 |
| Arhgap15 | Rho GTPase activating protein 15 | -2,83037 |
| Pcdhga2 | protocadherin gamma subfamily A, 2 | -2,82696 |
| Slc8a1 | solute carrier family 8 (sodium/calcium exchanger), member 1 | -2,81667 |
| Nudt6 | nudix (nucleoside diphosphate linked moiety X)-type motif 6 | -2,80635 |
| Rab11fip4 | RAB11 family interacting protein 4 (class II) | -2,80139 |
| Gm527 | predicted gene 527 | -2,80139 |

**Table S9. Top 100 down-regulated DEGs in retrieved MSCs (56D CIOA vs 56D SHAM)**

| **14D Retrieved MSCs** | | | |
| --- | --- | --- | --- |
| **Upstream Regulator** | **p-value** | **Activation Z-Score** | **Expressed LogRatio** |
| IL1B | 2.21E-20 | 2.455 |  |
| TNF | 1.60E-18 | 0.983 |  |
| IFNG | 5.45-18 | 4.137 |  |
| TP53 | 3.50E-16 | 0.127 | -0.058 |
| APP | 2.86E-14 | 1.363 | -0.197 |
| STAT3 | 5.73E-14 | 0.048 | 0.098 |
| SIRT1 | 1.30E-13 | -1.450 | -0.002 |
| TGFB1 | 6.13E-13 | -2.264 | 0.125 |
| NFKBIA | 9.20E-13 | 2.345 | -0.142 |
| HIF1A | 1.40-12 | 2.156 | 0.293 |
| **56D Retrieved MSCs** | | | |
| **Upstream Regulator** | **p-value** | **Activation Z-score** | **Expressed LogRatio** |
| IFNG | 7.73E-14 | 1.900 |  |
| TCL1A | 6.77E-13 | 2.813 |  |
| STAT3 | 2.01E-12 | 2.398 | 0.383 |
| IFNAR1 | 7.18E-11 | -0.637 | 0.000 |
| TMEM173 | 3.37E-10 | 1.540 | -0.635 |
| STAT1 | 8.01E-09 | 0.448 | -1.083 |
| IL1B | 8.23E-09 | 4.193 | 3.533 |
| OSM | 4.46E-08 | 3.098 |  |
| IFN alpha/beta | 9.52E-08 | -0.524 |  |
| IRF3 | 9.52E-08 | -1.292 | -0.229 |
| CCR2 | 1.03-07 | 2.276 | 1.569 |
| PTGER4 | 3.37E-07 | 1.038 | 0.000 |

**Table S10. Predicted upstream regulators for early and late OA retrieved MSCs with the corresponding p-value, activation z-score and the expressed log ratio when identified as DEGs.**

| Gene Ontology Enrichment of 14D Differentially Expressed Genes | | | | | | | | |
| --- | --- | --- | --- | --- | --- | --- | --- | --- |
| GO Biological Process | **P-value** | | | **Adjusted p-value** | | **Odds ratio** | | **Combined score** |
| Regulation Of Smooth Muscle Cell Migration (GO:0014910) | 0.000006146 | | | 0.01045 | | 8.91 | | 106.91 |
| Extracellular Matrix Organization (GO:0030198) | 9,75E-08 | | | 4,97E-04 | | 3.50 | | 80.71 |
| Extracellular Matrix Disassembly (GO:0022617) | 2,05E-04 | | | 0.0005237 | | 4.70 | | 72.37 |
| Positive Regulation Of Smooth Muscle Cell Migration (GO:0014911) | 0.0006129 | | | 0.1840 | | 9.16 | | 67.79 |
| Establishment Of Apical/Basal Cell Polarity (GO:0035089) | 0.001347 | | | 0.2370 | | 7.64 | | 50.48 |
| GO Molecular Function | **P-value** | | | **Adjusted p-value** | | **Odds ratio** | | **Combined score** |
| Insulin-Like Growth Factor II Binding (GO:0031995) | 0.002542 | | | 0.4876 | | 9.82 | | 58.66 |
| Collagen Binding (GO:0005518) | 0.00001203 | | | 0.006924 | | 4.85 | | 54.9 |
| Integrin Binding (GO:0005178) | 0.000002850 | | | 0.003280 | | 3.90 | | 49.79 |
| Adenyl Nucleotide Binding (GO:0030554) | 0.01051 | | | 1.000 | | 6.25 | | 28.46 |
| Adenyl-Nucleotide Exchange Factor Activity (GO:0000774) | 0.01051 | | | 1.000 | | 6.25 | | 28.46 |
| GO Cellular Component | **P-value** | | | **Adjusted p-value** | | **Odds ratio** | | **Combined score** |
| Platelet Dense Granule Lumen (GO:0031089) | 0.0002255 | | | 0.1006 | | 8.18 | | 68.70 |
| Filopodium Membrane (GO:0031527) | 0.001347 | | | 0.2003 | | 7.64 | | 50.48 |
| Pseudopodium (GO:0031143) | 0.005713 | | | 0.4246 | | 7.64 | | 39.44 |
| Platelet Dense Granule (GO:0042827) | 0.001777 | | | 0.1982 | | 5.45 | | 34.54 |
| Mitotic Spindle Midzone (GO:1990023) | 0.007898 | | | 0.4403 | | 6.87 | | 33.27 |
| Gene Ontology Enrichment of 56D Differentially Expressed Genes | | | | | | | | |
| GO Biological Process | | **P-Value** | **Adjusted P-Value** | | **Odds Ratio** | | **Combined Score** | |
| Leukocyte Aggregation (GO:0070486) | | 0.00006036 | 0.1540 | | 8.91 | | 86.53 | |
| Amino Acid Import Across Plasma Membrane (GO:0089718) | | 0.001184 | 0.5035 | | 7.13 | | 48.02 | |
| Regulation Of Macrophage Differentiation (GO:0045649) | | 0.0002761 | 0.2348 | | 5.75 | | 47.16 | |
| Positive Regulation Of Macrophage Differentiation (GO:0045651) | | 0.002218 | 0.4715 | | 6.23 | | 38.10 | |
| T-Helper Cell Lineage Commitment (GO:0002295) | | 0.002218 | 0.4526 | | 6.23 | | 38.10 | |
| GO Molecular Function | | **P-Value** | **Adjusted P-Value** | | **Odds Ratio** | | **Combined Score** | |
| Cxcr Chemokine Receptor Binding (GO:0045236) | | 0.00002116 | 0.02436 | | 5.87 | | 63.16 | |
| Basic Amino Acid Transmembrane Transporter Activity (GO:0015174) | | 0.0001595 | 0.09179 | | 6.23 | | 54.51 | |
| Nadph Binding (GO:0070402) | | 0.0005897 | 0.2263 | | 6.23 | | 46.36 | |
| Azole Transmembrane Transporter Activity (GO:1901474) | | 0.008560 | 0.6568 | | 6.23 | | 29.68 | |
| Phospholipase Activator Activity (GO:0016004) | | 0.008560 | 0.6158 | | 6.23 | | 29.68 | |
| GO Cellular Component | | **P-Value** | **Adjusted P-Value** | | **Odds Ratio** | | **Combined Score** | |
| Rad51b-Rad51c-Rad51d-Xrcc2 Complex (GO:0033063) | | 0.01409 | 1.000 | | 5.34 | | 22.78 | |
| Atp-Binding Cassette (Abc) Transporter Complex (GO:0043190) | | 0.02122 | 1.000 | | 4.68 | | 18.02 | |
| Replication Fork (GO:0005657) | | 0.01503 | 1.000 | | 2.88 | | 12.08 | |
| Primary Lysosome (GO:0005766) | | 0.04029 | 1.000 | | 3.74 | | 12.01 | |
| Scf Ubiquitin Ligase Complex (GO:0019005) | | 0.009876 | 1.000 | | 2.31 | | 10.66 | |

**Table S11. Top 5 Gene ontology enrichment terms for differentially expressed genes in early and late OA retrieved MSCs.** For each data set of DEGs we identified the enriched biological processes, molecular functions and cellular components with the corresponding p-value, adjusted p-value, odds ratio and combined score.


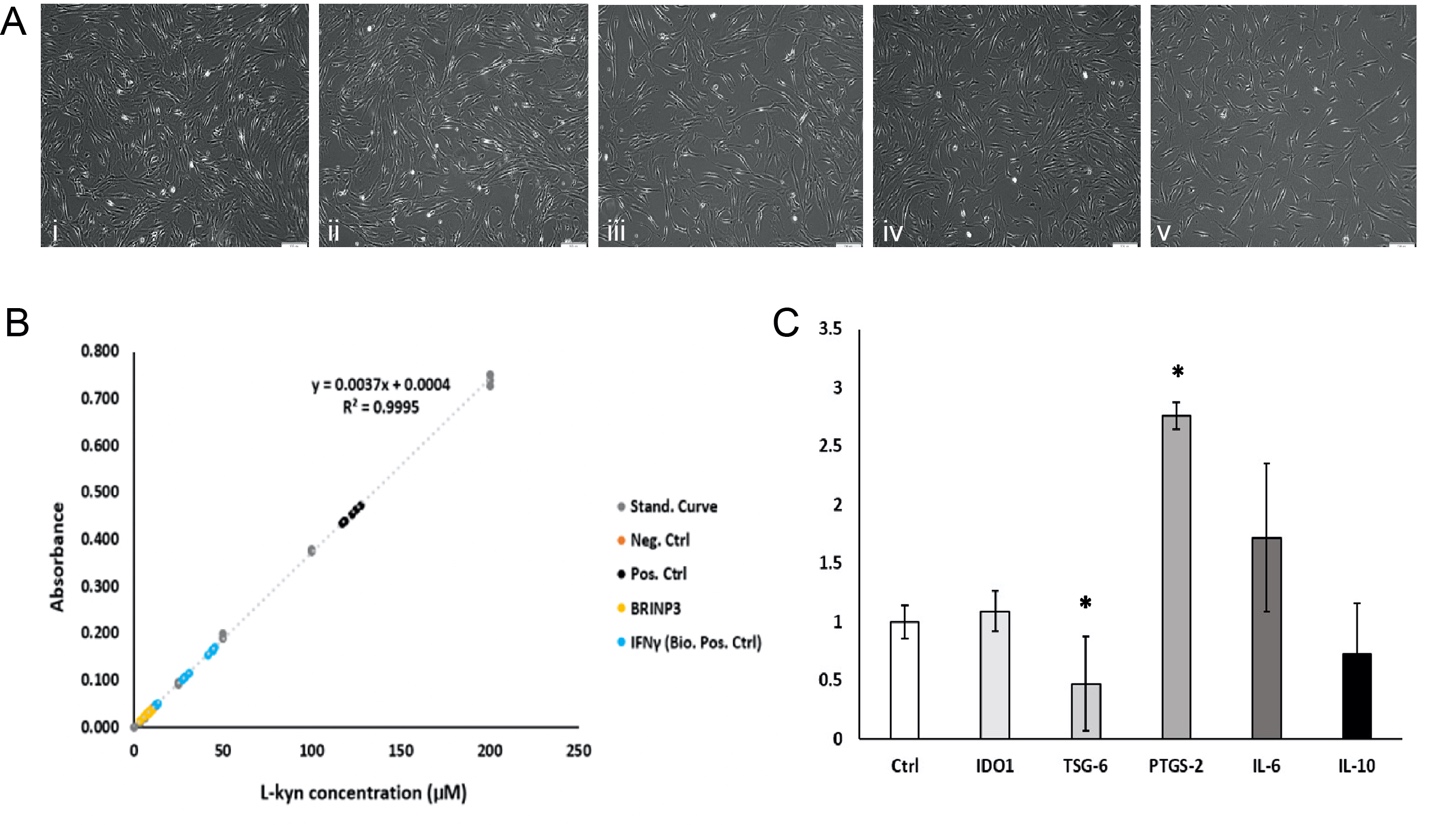


**Fig. S10. BRINP3 as licensing factor for MSCs.** (A): Morphology of control (i) and licensed hBM-MSCs (ii-v) with BRINP3 at corresponding concentrations of 10, 25, 50 and 100 ng/ml. All images were taken after 96 h of cultures. (B): IDO assay. The evaluation of the IDO production was assessed on the supernatants of cells licensed with IFNγ as positive control and BRINP3, after 96 h of cultures (72 h of exposure to the licensing factors). (C): qRT-PCR expression of anti-inflammatory genes after licensing with BRINP3 at 50 ng/ml. In each graph different expression results of five anti-inflammatory genes results are showed (IDO1, TSG-6, PTGS-2, IL-6, IL-10). Sample defined as “Ctrl” refers to unlicensed cell cultures used as controls. Results are represented as mean values ± SD of 3 independent experiments (3 donors). Ct values have been normalized to an unlicensed control and to a housekeeping gene (ACTB) prior the calculations of the ΔΔCt values. Results were considered significant when p-value was below 0.05.

| Missing Gene | Matching Synonym |
| --- | --- |
| Ecrg4 | 1500015O10Rik |
| wisp2 | Ccn5 |
| Siglec10 | Siglecg |
| Ero1b | Ero1lb |
| Nov | Ccn3 |
| Ctgf | Ccn2 |
| Chi3l1 | Chil1 |
| Txn | Txn1 |
| Cyr61 | Ccn1 |
| Plac9 | Plac9b |
| C4bpa | C4bp |
| Dnase2b | Dnase2a |
| Lilrb3 | Pirb |
| Mtnd4 | mt-nd4 |
| Mtnd6 | mt-nd4 |
| Il4r | Il4ra |
| Gsdmdc1 | Gsdmd |
| Pigy | Pyurf |
| Mic13 | 2410015M20Rik |
| 2610524H06Rik | 1500011B03Rik |
| Tmem167a | Tmem167 |
| Gpi | Gpi1 |
| Ca12 | Car12 |
| Ugt1a6 | Ugt1a6a |
| Kitlg | Kitl |
| Ca11 | Car11 |

**Table S12: Mismatched gene names between Ensemble annotation and symbols from expression data**


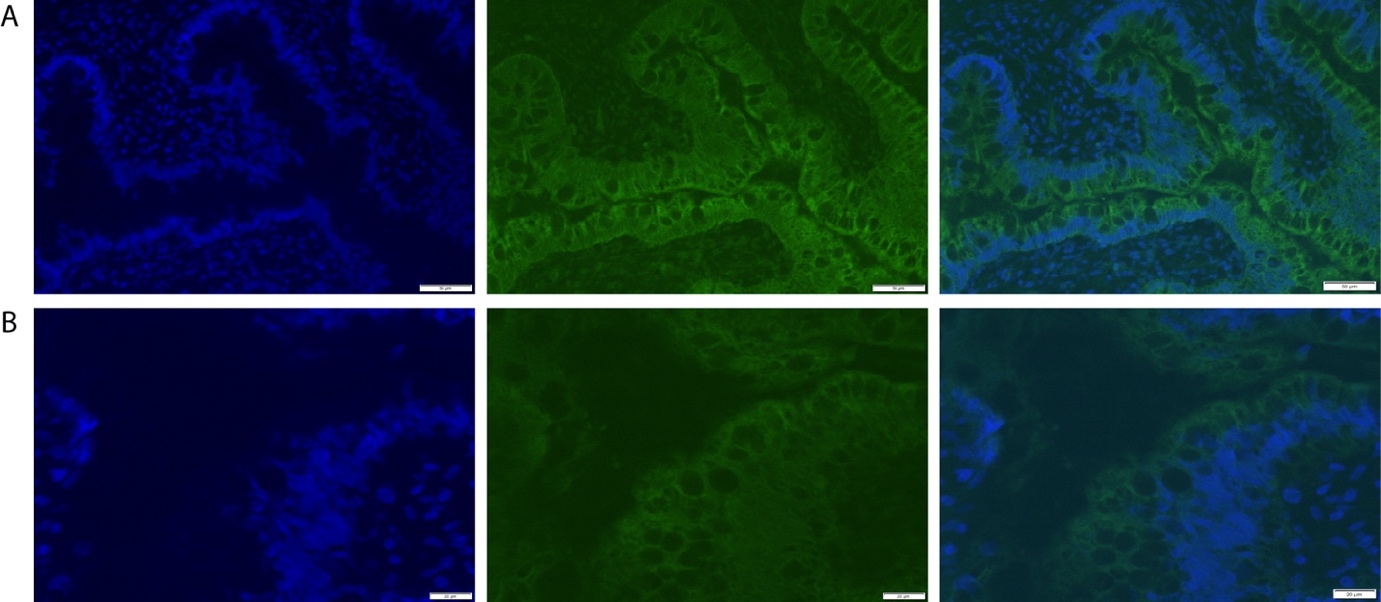


**Fig. S11.** Expression of BRINP3 in human teratomas at low magnification (A) and high magnification (B) images for antibody validation
